# Factors influencing technology use among low-income older adults: A systematic review

**DOI:** 10.1016/j.heliyon.2023.e20111

**Published:** 2023-09-13

**Authors:** Diana Yian Lian Chan, Shaun Wen Huey Lee, Pei-Lee Teh

**Affiliations:** a School of Business, Monash University Malaysia, Jalan Lagoon Selatan, 47500, Bandar Sunway, Selangor, Malaysia; b School of Pharmacy, Monash University Malaysia, Jalan Lagoon Selatan, 47500, Bandar Sunway, Selangor, Malaysia; c School of Pharmacy, Taylor's University Lakeside Campus, Jalan Taylor's, 47500, Subang Jaya, Selangor, Malaysia; d Gerontechnology Laboratory, Monash University Malaysia, Jalan Lagoon Selatan, 47500, Bandar Sunway, Selangor, Malaysia

**Keywords:** Low-income older adults, Technology use/ adoption, Systematic literature review, Social cognitive theory, Maslow's hierarchy of needs

## Abstract

As the world's aging population increases, leveraging technology to support aging is proving advantageous. Notably, technology adoption studies among older adults have received increasing scholarly attention, but findings from these studies do not reflect the context of low-income older adults. Studies focusing on low-income older adults were relatively few and it remains unclear which factors influence this group's technology use. This systematic review aims to synthesize findings on factors influencing technology use among low-income older adults to provide directions and opportunities for future research in information systems. Observing the literature through the lens of Social Cognitive Theory, we identified avenues for future research and further integrated the framework with Maslow's hierarchy of needs to elucidate the phenomenon. Findings from this systematic review suggest that both personal and environmental factors, such as cognitions, affects, sociodemographic characteristics, technological and social environment are significant predictors of technology use among low-income older adults. Specifically, factors related to accessibility and affordability, such as income, perceived cost, and accessibility to technology are salient in a resource-limited setting. More importantly, the technology usage behavior elucidate the embeddedness of fundamental human needs which plays a central role underlying technology use among this segment. However, more research is needed to understand the interaction between person, environment and behavior determinant shaping technology use among low-income older adults from diverse economic and cultural setting. This study also sheds light on disciplinary gaps and the lack of investigations anchored on theoretical foundations, and suggests avenues for future research and implications for practice.

## 1 Introduction

The United Nations has called for researchers, policymakers, service providers and leaders of organizations to harness our strengths to improve the lives of older adults following the Decade of Healthy Ageing (2021–2030) global initiative [1]. Study of older adults’ technology adoption has been an information systems (IS) research focus in recent years [2,3], wherein signifies the important role of IS research and development in contributing to the aging population. The use of technologies has been shown to be able to enhance the lives of older adults by providing support for aging in place [4,5], independent living [6], social participation [7,8], and improving quality of life [9] for older adults.

Nevertheless, IS research focusing primarily on the low-income segment is limited [10]. Low-income older adults are regarded as a vulnerable population that faces multiple and intersecting challenges to aging well in the community. Besides experiencing different needs and concerns resulting from biological changes (i.e., physical and cognitive functions) of aging, they are also susceptible to socioeconomic challenges, such as insufficient income to cover daily expenses and limited resources to manage unexpected changes in their health or living environment. Consequently, low-income older adults are at risk for poor mental and physical health [11], social isolation and loneliness [12,13] and in some cases, they have to rely on social services or community-based care and assistance to live independently [14,15] in their own homes to avoid institutionalized care. Leveraging technologies has the potential to support aging and improve health and wellbeing [7,16] for this segment. For instance, enhancing low-income older adults' use of information and communication technologies (ICT) can provide them with access to relevant health information and services available to them, maintaining connections to their families and communities, and participating in meaningful activities. Similarly, the use of telehealth and remote monitoring technologies may enhance older adults’ self-management of chronic illness, sustain communication and coordination with relevant parties involved in their care, and provide them with the possibility of arranging services online. This is an important way forward because technology-mediated approaches could potentially support old-age dependency, reduce the cost of long-term care [17] and provide sustainable solutions for improving the lives of low-income older adults. The ultimate goal is to ensure that the Sustainable Development Goals (SDGs) are met for all segments of society, at all ages, with a particular focus on the most vulnerable groups.

Despite technology having many potential benefits to improve the lives of low-income older adults, the socially and economically disadvantaged are generally overlooked in technology design and development, such as in the case of eHealth design [18] and are at risk of becoming digitally marginalized, leading to a potential widening of health disparities. In some cases, this group is discounted as “non-adopters” [18] because of their known sociodemographic barriers, such as age, education, ethnic origins and income, yet they are the people who may need and benefit the most from technological interventions. At present, studies focusing on low-income older adults are still relatively few and it remains unclear which factors influence their use of technology [19,20] – we could not locate any systematic review study on this specific segment of society. Although many systematic reviews of factors influencing older adults' use of technology exist in the literature [2,[21], [22], [23]], the findings do not reflect the context of low-income and thus limit its generalizability and applicability to this population. In fact, studies have shown digital divide by socioeconomic status within the older adult population [24]. In order to better inform and advance research on low-income older adults’ use of technology, we need to begin by taking stock of the current status of the literature, consolidating what we already know to provide a clearer direction for future research. Against this backdrop, a systematic review is timely and needed to draw multidisciplinary focus on this research area.

This review aims to answer the following questions: (1) Which factors influence technology use by low-income older adults? (2) Which theories and frameworks have been employed in prior research on factors influencing low-income older adults' use of technology? (3) What avenues for future research emerge from the extant literature? In this review and synthesis, we do not make distinctions between the types of technologies or evaluate their usage level or the consequences of their use; instead, we are aiming to synthesize as much relevant knowledge from the literature as possible regarding the factors influencing use, and to demonstrate the relevant factors in an integrative framework. Specifically, we drew upon Social Cognitive Theory (SCT) [25,26] and further integrated Maslow's hierarchy of needs [27,28] to develop an integrative framework that summarizes the factors related to low-income older adults' technology use behavior. This systematic review reveals what is known and unknown and outline future research agenda.

### 1.1 Definitions of key terminologies

This study focuses on the factors influencing technology use among low-income older adults. In the context of our review, factors are determinants or variables associated with the use of technology among low-income older adults.

#### 1.1.1 Low-income older adults

The United Nations defines older adult as individuals age sixty and above [1]. Nevertheless, because this study's ultimate goal is to inform technological interventions designed for the low-income older adult group, this review considers older adults as individuals starting from the age of fifty years and older based on two contextual propositions: (i) the gap in life expectancy between the richest one percent and poorest one percent of individuals was 14.6 years for men and 10.1 years for women in the United States between 2001 and 2014 [29], and low-income older adults may need technological interventions related to these aging and health issues earlier in the aging process than the general population of older adults; (ii) the ‘older adults’ age classification of fifty years and above is commonly observed in large longitudinal studies on aging, such as the Health and Retirement Study in the United States 2017 [30] and the English Longitudinal Study of Ageing (ELSA) 2020 [31]; and (iii) consistent with the ‘older adults’ age range adopted in existing systematic literature reviews on factors influencing technology use by older adults [23,32,33] and empirical studies concerning low-income older adults, such as healthcare access and food insecurity [34], interventions [35,36], and Internet use [37].

How ‘low income’ is defined varies in countries and studies, including whether a more objective dollar amount is used, or a more subjective marker of financial burden or distress [38]. For instance, the World Bank classifies countries as low-income based on income per capita below USD $1,085, and defined people living in poverty as those living below the global poverty line of USD $2.15-$6.85 per person per day [39]. However, what constitutes individuals of low-income households were not specified and there were no grounds to suggest that every person in a low-income country is of low-income. The OECD defines low-income households as households in the bottom quintile of the (net) income distribution, or individuals with a net income below fifty percent of median income of the total population [40]. The approach to defining low income in this study is in line with the recommendations by Jones et al. [38], that “the operationalization of low-income likely most importantly depends on the research questions and hypotheses” because broad heterogeneity exists in the construct of ‘low income’ [38]. It is important to note that providing a comprehensive understanding of ‘low income’ or calculating income for policy evaluation is not the main focus of this study. Given that there is no straightforward standard and definition of ‘low income’, this study does not limit itself to a single indicator of ‘low income’. A broad definition based on the above discussion is applied, with two goals: (i) to capture a broad and diverse sample of low-income older adults; and (ii) to take an inclusive approach so that more low-income segment is accounted for. The characteristics of the low-income older adults captured in this review are discussed in the following section.

#### 1.1.2 Use of technology

In this paper, the use of technology is defined as the adoption of technology whether a priori (i.e., intention to use or acceptance) or posteriori of the interaction with technology (i.e., actual use; continuous use) [41,42]. This study does not aim to delineate different phases of technology adoption; instead, the goal is to synthesize relevant factors of technology use. Therefore, the terms ‘technology use’ and ‘technology adoption’ are used interchangeably in this paper. This present review also adopts a broad definition of technology. It is important to note that the present review is not focused on a specific type of technology or the evaluation of level of acceptability or level of usage of a particular technology, but focuses mainly on providing an overview of the current literature concerning technology use among the low-income older adults.

This paper is structured as follows. We begin by discussing the theoretical foundation, describing our literature search and identification procedures followed by a brief overview of the status of extant research on factors influencing low-income older adults' technology use. Following this, we provide a more detailed description on the factors related to low-income older adults’ technology use, synthesized and mapped out in our integrative framework. Finally, we discuss directions and opportunities for future research and the contributions and limitations of this study.

## 2 Theoretical background

Given that multi-facet demographic-related factors may influence technology use among the low-income older adults, it would be useful to employ a *general* theory to map the factors observed in studies to present an integrative framework, rather than an existing model which was developed based on the broader older adult population such as Senior Technology Acceptance Model (STAM) [43] or Unified Theory of Acceptance and Use of Technology (UTAUT) 2 [44]. Hence, this study builds upon an existing Social Cognitive Theory-based framework on computer use by older adults in Wagner et al.’s [3] systematic review on computer use by older adults and extends it to the context of technology use among the low-income segment.

Developed by Bandura, SCT posits that one's behavior is shaped and controlled by two main determinants: personal and environmental elements [25,26] and these three determinants of person, environment and behavior influence one another in a triadic reciprocal relationship [45]. In other words, human behavior is regulated by an interplay of self-generated and external sources of influence [46] and the two major cognitive forces guiding an individual's behavior are outcome expectations and self-efficacy [46,47]. In the technology adoption context, personal factors refer to any attributes of a low-income older adult, such as sociodemographic characteristics, cognitions (i.e., cognitive evaluations of the likely consequences of performing a task and self-efficacy) and affects (i.e., emotions) [3]. Distinct from Wagner et al.’s [3] SCT-based framework which refers environment to the computer system, environment in the present review encompasses any social, technological, physical and organizational environmental characteristics. Behavior refers to the actions of using a technology or intention to use a technology.

Employing SCT as a theoretical lens to demonstrate factors influencing low-income older adults’ technology use in an integrative framework is appropriate for several reasons. First, since SCT provides a basis for behavior intervention strategies [47], the interaction between the determinants illustrate means through which technology use can be encouraged among low-income older adults. Drawing upon SCT allows researchers to view the factors through a theoretical lens to organize them into a conceptual framework [3] to understand to which extent the “person”, “environment” and “behavior” relationships have been investigated and the avenues for future research. Second, the inclusivity of SCT was comprehensive enough to allow us to capture and integrate the multi-facetted factors identified in the literature, originating from multiple disciplines and with varied research foci. SCT is used widely in many disciplines, such as psychology, education, public health [48]; management and IS [49].

## 3 Method

### 3.1 Literature search and identification

This systematic review was conducted following the Preferred Reporting Items for Systematic Reviews and Meta-Analyses (PRISMA) 2020 guidelines [50] (OSF Ref: MD2QH) https://doi.org/10.17605/OSF.IO/MD2QH.

#### 3.1.1 Search strategy

We searched for empirical studies in the English language that describe technology use among older adults. Following the sampling strategy of subject specific and multidisciplinary databases in prior systematic review [51], we identified databases for our literature search using four steps. First, for the review's focus on older adult populations, we selected EBSCOhost which covers database, such as AgeLine with research focusing exclusively on the population aged 50+. To cover the aspect of low-income populations, we included EBSCOhost which covers Social Sciences Index Retrospective which focuses on research in social sciences. Third, to cover research on technology adoption, we also enlisted EBSCOhost which covers Business Source Complete focusing on information systems research, and MEDLINE which includes life sciences, public health, and healthcare-related literature; PubMed which covers behavioural sciences literature including medical informatics; and PsycINFO which contains literature of psychological, social, behavioral, and health sciences. Fourth, we included multidisciplinary databases to ensure our search was thorough. This included ProQuest Central, and Web of Science. These steps yielded a total of five databases, namely EBSCOhost, ProQuest Central, Web of Science, PubMed, and PsycINFO. Electronic searches were performed from database inception to October 31, 2022. To reduce the likelihood of missing relevant studies, we used broad search terms and included a wide range of keywords in order to find as many potentially relevant articles as possible. The search terms used are listed in Table 1. This exercise was supplemented with manual searches of reference lists of retrieved articles and searches in Google Scholar to identify relevant gray literature (e.g., conference papers, and proceedings), and articles not found in the databases previously mentioned.

**Table 1:** Search terms used.

	Category	Search terms
1	older adult	older adult OR older people OR seniors OR elderly OR aging OR ageing
2	low-income	low-income OR bottom-of-pyramid OR low socioeconomic status OR poverty
3	technology	technology OR gerontechnology OR mobile application OR mobile app OR mobility application OR assistive technology OR ride-hailing application OR technology-mediated
4	adoption	acceptance OR adoption OR behavioral intention OR behavioral intention OR use
5		1 AND 2 AND 3 AND 4

#### 3.1.2 Study selection and quality assessment

Retrieved articles were independently screened by title and abstracts by two reviewers. Studies were included if: (i) participants are low-income older adults aged fifty years or above living in the community (i.e., not living in a long-term care institution). Samples might include non-older adults but reported findings of older adults distinctively; (ii) empirical studies that investigated, but were not limited to, factors related to low-income older adults' use of technology; (iii) research articles, conference papers and proceedings. The screening and selection of the studies was a three-step process. First, in the initial screening, articles generated in the database searches were checked for relevance based on whether title and abstract were related to factors influencing low-income older adults' technology use. Studies with no relevant data or information of interest to the review question were excluded. Second, articles included from the initial screening were read in full. Third, based on the relevant articles, forward and backward searches were performed. Forward searches were conducted using Google Scholar, especially for articles cited often or covering aspects very different from other articles. Backward searches were done by studying the reference list of the articles included. Any disagreements regarding the inclusion and exclusion of an article between the two authors were resolved by discussion until consensus was reached. In case of doubt, three authors discussed the selection. We subsequently assessed the quality of the studies following the method in Peek et al.’s [4] systematic review: adopting the Critical Appraisal Skills Program (CASP) [52] for qualitative studies and the Health Evidence Bulletins Wales (HEBW) [53] checklist for quantitative studies. Ratings of each article were assessed using Peek et al.’s [4] template, and discrepancies were resolved through discussion and consensus. None of the studies were excluded on the basis of quality assessments.

### 3.2 Data extraction and analysis

This review followed the PRISMA statement [50] when applicable, with consideration of the specific context of low-income older adults. The following data were extracted for each article into Excel templates developed by the authors: authors; year of publication; title; journal; research question and objectives; sample background including health condition; personal or household income; education; country; sample size; age range; study design; type of technology; theories and framework; factors associated with technology use; technology use behavior (i.e., use or intention to use, usage); test or analysis conducted; and conclusion. Detailed data regarding the factors associated with technology use, such as the odds ratios or percentages; p-value of regression analysis; positive or negative impact towards technology use were documented for quantitative studies. For qualitative studies, we documented themes, subthemes or subcategories, and data excerpts of how low-income older adults’ perspectives, experiences and perceptions influence their use and intention to use technology.

After extracting data, we coded both quantitative and qualitative factors by grouping factors of similar concepts in the same cluster. For qualitative data, whenever the themes represent a concept too broad to depict a distinct factor, the subthemes or subcategories would be coded instead. We adopted the coding method following prior systematic review [54] using existing factors mentioned in the article as codes, and develop a new code each time an existing code could not capture a specific factor. Through several iterative process and discussions, we aggregated the codes and generalized into broader concepts of factors and domains, and drew upon the theoretical lens of Social Cognitive Theory [25,26] to synthesize the factors and domains in a conceptual framework to present an overview of factors influencing technology use among low-income older adults. Statistical analyses were not conducted to test causes of heterogeneity among study results, assess the robustness of synthesized results, assess the risk of bias for missing results, or assess the certainty of evidence because these items were not applicable to the objective of this study. There were no amendments to information provided in the protocol.

## 4 Results

Results are discussed in Section 4, 5. Section 4 gives a general overview of the samples, the participant characteristics in the included studies, theories and frameworks employed. Section 5 describes the factors influencing technology use illustrated in an integrative framework.

### 4.1 Overview of empirical studies

**Fig. 1:**
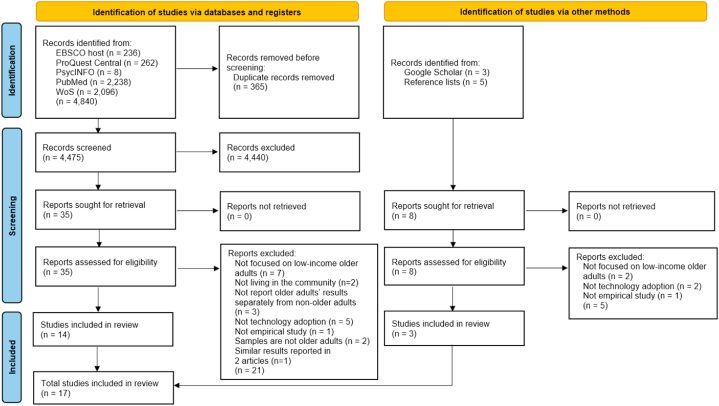
PRISMA 2020 flow diagram of an overview of literature search and identification process.

After duplicate records from database searches were removed, 4475 records were screened, based on their titles and abstracts, resulting in thirty-five articles to be retrieved and further assessed for eligibility. Supplemental manual searches of reference lists of retrieved articles and Google Scholar yielded eight articles. Scrutiny of full texts excluded twenty-one articles from database searches and five articles from the supplemental searches. In total, seventeen articles were retained and considered in the present review (Appendix A). The PRISMA diagram (Fig. 1) presents the inclusion and exclusion procedures performed, combining the articles from the databases and those from reference lists and Google Scholar.

### 4.2 Study characteristics

**Fig. 2:**
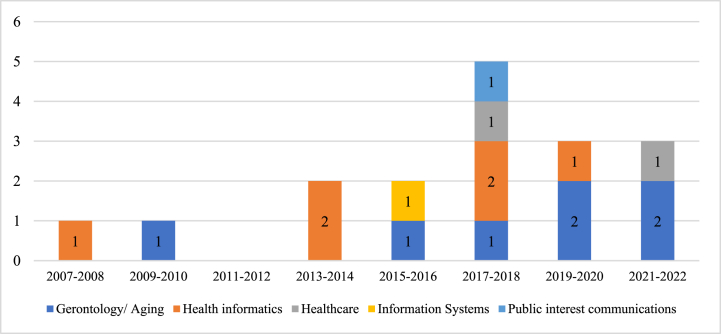
An overview of the number of studies conducted over time across disciplines.

**Fig. 3:**
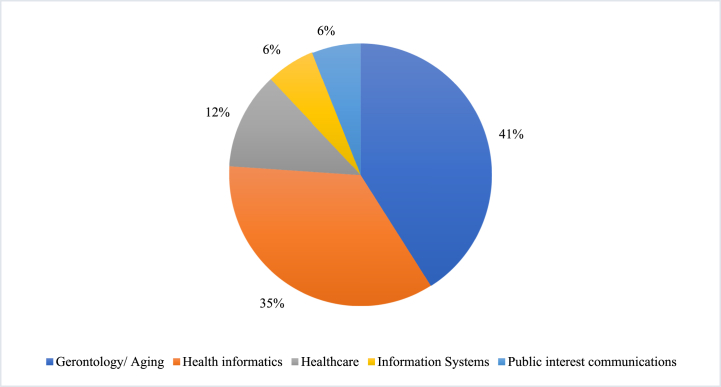
An overview of research trend of low-income older adults' technology use across disciplines.

To our knowledge, among the seventeen studies in our sample, no studies were conducted outside of the USA. Publication trends depicted that the study of technology use among low-income older adults has received fairly limited scholarly attention in the past decade. A steady increase in research can be observed and most papers were published between the years 2017 and 2018 (five articles). An overview of the number of studies conducted over time and across disciplines can be seen in Fig. 2. The majority of studies were published in the gerontology or aging (41%) and medical informatics (35%) literature, and the remaining were published in healthcare (12%), IS (6%), and public interest communications (6%) as illustrated in Fig. 3. This research trend suggests that this research foci received the most attention from the gerontology, aging and medical informatics fields, and was primarily aimed towards supporting the aging community, particularly through technology interventions to close the digital divide and reduce aging and health disparities among the low-income segment [55,56]. The most common types of technology in the studies reviewed are ICT-related (e.g., computer, mobile technology and the Internet) which constitutes 58.8%, followed by digital health technology (e.g., telemedicine, tele-psychotherapy, patient portal) at 23.5%, smart sensor technology (e.g., remote monitoring system) at 11.8% and artificial intelligence technology (e.g., Intelligent Voice Assistant (IVA) 5.9%, as depicted in Table 2. The limited distribution of types of technology focused upon suggests that the use of ICT was seen as the most basic technology to access other technology interventions while health-related technologies were aimed at reducing health disparities and to support independent living in an aging and low-income community. Both the survey and interview methods were commonly used in the low-income older adults’ technology adoption studies and the strands of research uncovered different but interrelated factors which are discussed in Section 5. The quality assessment of articles revealed that all the publications were considered to be of acceptable quality.

**Table 2:** Distribution of types of technology studied.

Type(s) of technology	Technology	Total
ICT	The Internet, computer, mobile phone, smartphone	10
Digital Health Technology	Telecare	1
	Patient portal	2
	Tele-delivered behavioral activation (Tele-BA)	1
Smart Sensor Technology	Sensor-based Remote Monitoring System	2
Artificial intelligence	Intelligent Voice Assistants (IVAs)	1
Total		17

### 4.3 Participant characteristics of studies

Samples in the studies included in our systematic review samples were low-income older adults who ranged between ages fifty and one hundred and six years (one study did not report the age range), with a total of 8623 older adults in all 17 studies. Among these studies, a wide range of low-income financial indicators were observed with annual income of less than USD $5000 on the lower end of the scale or less than 100% poverty level and below USD $35,000 or above 200% poverty level on the higher end of the scale [55,[57], [58], [59], [60], [61], [62], [63], [64], [65]]. Studies also reported income-to-needs ratio (amount of income relative to the cost of living) below 1.21 [56]; and income in the past 30 days of USD $0-$1150 [66] (four studies did not report the income range or percentage of poverty level) [[67], [68], [69], [70]]. Notably, the sample characteristics were diverse across the studies, such as: homeless-experienced [66]; homebound due to mobility impairment [56]; US Medicare beneficiaries [60]; patients at health centres or urban and rural clinics serving low-income populations [55,57,70]; immigrants [62,67]; immigrants living in subsidized independent living residential [58,59]; older adults living with a disability, from subsidized public apartments [64]; older adults living in public senior housing facilities [63,69]; minorities residing in an affordable housing complex [19]; marginalized older adults from different racial or ethnic backgrounds [68]; members of a community centre located in one of the poorest neighbourhoods [65]; and older adults receiving home-delivered meals from aging-service agency [61]. In general, the population studied had cognitive impairment, and impairments in executive function [66]; were being treated for a chronic disease (diabetes, hypertension, dyslipidemia, or cardiovascular disease) [55,57]; had an average of three chronic illnesses, diagnosis of depression and anxiety [56]; had chronic health conditions, such as arthritis, hypertension, diabetes, chronic pain and asthma [19,58,59]; and were mobility impaired, homebound, having moderately severe to severe depressive symptoms [61].

### 4.4 Theories and frameworks

Only one quarter of studies (*n* = 4) employed theories and frameworks in their studies, namely the Technology Acceptance Model (TAM) [41], Person-Environmental Interaction Model (P-E) ([71], and Diffusion of Innovation (DOI) Theory [72]. Table 3 summarizes the theories and frameworks adopted and how they were applied in the studies. Of four studies, half used theories to explain how specific factors influence low-income older adults' technology use. For instance, TAM was employed to understand how and why perceived usefulness and perceived ease of use influence Internet and computer use [65], and the P-E Interaction Model was used to provide a conceptual framework to explain how personal and environmental factors influence the use of a patient portal and the Internet [55,57]. However, the applications of theories and models were constrained in the other two studies. For instance, Arcury et al. (2017) [57] examined how older adults' characteristics are associated with perceived usefulness and usability but did not investigate their effects on technology use; and Diffusion of Innovation [72] was used as a backdrop to distinguish older adults who were early adopters and non-adopters but did not examine the existing DOI technology acceptance constructs in the study [62]. The number of studies that conceptualized upon theoretical foundations and discussed how theories were applied in the studies suggest that theoretical investigations of the factors influencing low-income older adults’ technology adoption was less than thorough in the extant literature.

**Table 3:** An overview of the theoretical foundations of low-income older adults’ technology adoption studies.

Theory/Framework	Description	Application	Study
Technology Acceptance Model (TAM) [41]	This model suggests that two key beliefs (i.e., perceived ease of use and perceived usefulness) leads to attitude, followed by intention and behavior.	The study examined how older adults' characteristics are associated with perceived usefulness and usability. However, TAM was not used to explain how perceived usefulness and perceived ease of use influence technology use behavior.	[55,57]
		The model was used to understand how and why perceived usefulness and perceived ease of use influence technology use behavior.	[65]
Person-Environmental Interaction Model [71]	This model suggests that behavior and well-being is largely influenced by the degree of fit between personal needs and environmental resources.	The model was used to provide a conceptual framework to explain how personal and environmental factors influence the use of technology.	[55,57]
Diffusion of Innovation [72]	This theory seeks to explain how, why, and at what rate people adopt innovations (e.g., technologies) and suggests that there are five adopter categories (i.e., innovators, early adopters, early majority, late majority, and laggards).	The theory was used as a backdrop to distinguish older adults who were early adopters and non-adopters. However, existing technology acceptance constructs drawing upon this theory (i.e., ease of use, relative advantage, values and preferences, compatibility, complexity, and trialability [42] were not applied in the investigation.	[62]

## 5 An integrative framework of factors related to low-income older adults’ technology use

Drawing upon the premise of SCT [25,46] and extending Wagner et al.’s [3] framework, we organized our findings in an integrative framework to illustrate factors influencing technology use among low-income older adults based on the relationships observed in the reviewed literature. Given the embedded fundamental needs motivating technology use uncovered in the literature, we further integrated SCT with Maslow's hierarchy of needs [27,28] to elucidate the phenomenon and provide a more holistic perspective of the explicit and implicit background factors driving technology use among this segment. Empirical evidence also showed that income plays a key role in the satisfaction of physiological needs [73], which assumed expenditure priority for someone who has limited disposable income [10]. For instance, one facing limited financial resources for daily essentials may be more concerned with physiological needs, such as hunger and shelter, and needs such as esteem and self-actualization can be suppressed when immediate physiological needs are salient [74]. Therefore, in our attempt to synthesize our findings, integrating Maslow's hierarchy of needs [27,28] provides contextual understanding of the present framework which highlighted the embeddedness of fundamental human needs plays a central role underlying technology use among this segment. Maslow's hierarchy of needs [27,28] is a taxonomy of fundamental human motivation developed with the idea of a cognitive hierarchy of human needs, that some needs take precedence over others, which in turn take precedence over others. The five layers of fundamental needs are as follows: physiological, safety, love and belonging, esteem, and self-actualization. Maslow's definition of physiological needs highlighted the needs for oxygen, air and food essential for human survival [27]. In the context of low-income older adults, physiological needs encompass shelter, food, and income that pay for these needs. Safety needs are the inherent search for security, protection and stability dealing with threats. Safety needs include dimensions, such as health, and well-being [75]. Love and belonging needs refer to the social needs of having close interpersonal relationships, involves giving and receiving affection, enjoying friendship and companionship in connecting with people. Esteem needs are the desire for high sense of self-worth; it involves self-esteem, and respect from others [75], giving a sense of being valuable, useful, and necessary in society [28]. Self-actualization needs refers to reaching one's purpose in life, the desire of realizing one's full potential, and reaching one's ideal life, as described by Maslow, ‘‘What a man can be, he must be” [28].

**Fig. 4:**
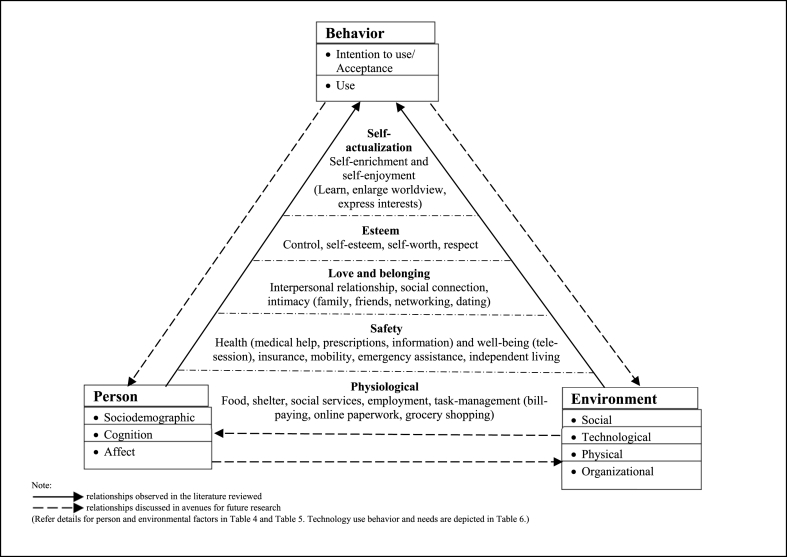
An integrative framework of factors related to low-income older adults’ use of technology.

This integrative framework offers an overview of the explicitly identified factors and implicit background motivation of fundamental human needs related to technology use among low-income older adults (Fig. 4). This SCT-based framework shows the triadic reciprocal relationship between all three determinants of person, environment and behavior [45]. Viewing the literature through the lens of SCT revealed the directions of relationship which have been observed in the reviewed studies and the opportunities for future research which are discussed in the following sections.

### 5.1 Personal factors

SCT posits that cognitive and other personal factors, such as sociodemographic characteristics, constitute personal determinants that account for shaping human behavior [25]. Personal determinant has been extensively studied in the literature as a key determinant associated with technology use among low-income older adults. The three personal domains that emerged from analysis are: (i) an individual's sociodemographic characteristics; (ii) cognitive evaluations; and (iii) affective responses. Personal factors influencing technology use among low-income older adults are depicted in Table 4. Among the domains, cognitive evaluations and affective responses emerged as significant factors regarding technology use. Among the sociodemographic factors, age, ethnicity, education, and income were the most consistently significant predictors whereas other sociodemographic factors yielded mixed findings. Most of the sociodemographic variables were tested quantitatively while most of the cognitive and affective factors emerged inductively from qualitative studies (refer details in Appendix B. Table B1 & Table B2).

**Table 4:** An overview of the personal factors related to technology use among low-income older adults.

Dimensions of Personal Determinant	Factor	Study
Sociodemographic characteristics	Age	[55,56,60]* [62]; n.s.
	Ethnicity	[55,56,60,66]* [57]; n.s.
	Education	[55,57,60,62]*
	Income	[55,56,60]* [62]; n.s.
	Experience	[57,62]*
	Cultural background +	[59] (q)
	Health knowledge and attitudes	[55]*
	Health condition/history	[55,57] n.s. [56,60,66];*n.s.
	Gender	[62]* [55,56,60]; n.s.
	Language	[56] n.s.
	Employment	[55] n.s.
Cognitive Evaluations	Perceived benefits (health)	[19]^ [61,64,70]; (q)
	Perceived benefits (mobility)	[68] (q)
	Perceived benefits (social participation)	[58,68,69] (q)
	Perceived benefits (self-actualization) +	[58,67,69] (q)
	Perceived benefits (monetary)	[69] (q)
	Self-efficacy	[62]* [60]; n.s. [68,69]; (q)
	Perceived usefulness	[65] (q)
	Perceived ease of use	[58,65,70] (q)
	Perceived risks +	[64,65,68,70] (q)
	Distrust +	[64,69] (q)
	Perceived cost +	[[68], [69], [70]] (q)
	Perceived threat to autonomy +	[58] (q)
Affective responses	Computer anxiety	[55,62]* [58,69]; (q)
	Aging anxiety	[62]*
	Interest	[70] (q)
	Freedom to express	[67] (q)
	Feeling productive and useful	[61] (q)
	Hope	[61] (q)
	Pride	[61] (q)
	Belonging	[62] n.s.

Note. *significant, n.s. nonsignificant, ^no correlation conducted, + aggregated and self-coded, (q) qualitative data (Refer Appendix B. Table B1 and Table B2 for details).

#### 5.1.1 Sociodemographic characteristics

In the present literature, investigations of the associations between sociodemographic characteristics and technology use were the most common, occurring in forty-one percent of the studies (*n* = 7) [[55], [56], [57],^59^,^60^,^62^,^66^] albeit not all sociodemographic factors show significant associations. Within the sociodemographic characteristic domain, eleven factors associated with low-income older adults’ technology use emerged: age, gender, ethnicity, language, education, employment, income, health knowledge and attitudes, health condition and history, cultural background and experience.

##### 5.1.1.1 Age; ethnicity; education; and income

Among sociodemographic factors, the effects of age [55,56,60], ethnicity [55,56,60,66], education [55,57,60,62], and income [55,56,60] were consistently significant across studies, suggesting that low-income older adults who are younger, of non-minority ethnicity (i.e., white), had higher education levels and higher incomes, were more likely to use technology. These findings also suggest that disparities exist even within the segment of low-income older adults due to age, ethnicity, educational attainment and level of income.

##### 5.1.1.2 Experience; cultural background; and health knowledge and attitude

A few studies found that experience [57,62], cultural background [59], health knowledge and attitudes [55] were significantly associated with technology use but the number of studies was fairly limited and no concrete conclusion could be drawn. For example, studies found that greater computer experience [62] and Internet use frequency [57] have positive effects on technology adoption. Further, low-income older adults with good health knowledge and attitude, such as adequate general health literacy, prefer not to rely only on their doctor's information for medical decision-making. They will have a number of health information sources and will have a greater tendency to use the Internet [55]. Conversely, older adults' technology adoption was sometimes hindered by their cultural background – this was the case among the older adult immigrants in the United States. This group were reluctant to adopt sensor-based remote monitoring systems in their homes for several reasons founded on values embedded in their culture, such as preferring death rather than prolonging a severely impaired life, values of filial obligation and this group would accept technology only when they lacked frequent family support. Social workers are needed as cultural navigators to help overcome linguistic and cultural barriers to technology use [59].

##### 5.1.1.3 Health condition/history

Health condition/history has been reported extensively in the literature but findings are inconclusive. For instance, a number of health factors that have been found to have significant negative effects on technology use are: cognitive impairment (using Modified Mini-Mental (3MS) Examination as instrument) [66]; executive function impairment (using Trail Making Test as instrument); moderate to high-risk amphetamine use [66]; and dementia and Alzheimer's disease [60]. Nevertheless, most of the studies yielded inconsistent findings regarding the impact of health condition or history on technology use. For instance, Raven et al. [66] found that older adults with activities of daily living (ADL) impairment (as compared to no ADL impairment) were less likely to use the Internet or mobile technology, whereas Choi & DiNitto [56,60] reported a non-significant effect. Similarly, Choi & DiNitto [60] reported that anxiety diagnosis was significant, and negatively associated with Internet and computer use, whereas conversely, these authors also found it not related to never having used the Internet or computers, or discontinued use [56]. Further, studies also report conflicting findings regarding the association between a depression diagnosis and technology use. For instance, Choi & DiNitto [60] reported that older adults diagnosed with depression were less likely to use the Internet or a computer; but these authors also found that older adults diagnosed with depression were less likely to have never used or discontinued use of the Internet or a computer [56]. Several studies examining health factors in terms of the number of chronic illnesses [60], medical conditions [56], chronic conditions measured using the Charlson Comorbidity Index [55,57], SF-12 Mental Component Score subscale [55], and Physical Component Score subscale [55] did not find any significant relationship between these health factors and technology use. Similarly, investigations of subjective measures of health such as self-rated good to excellent health or heavy drinking found the association with technology use to be non-significant [66]. These mixed findings were probably due to the varied aspects and measures of health employed in the respective studies to examine health conditions.

##### 5.1.1.4 Gender, language and employment

Of eleven sociodemographic factors, three have been found to be non-significant predictors of technology use: gender [55,56,60]; language [60]; and employment [55].

#### 5.1.2 Cognitive evaluations

Cognitive evaluations emerged as one of the two key predictors of technology use among low-income older adults. This finding demonstrates the underlying assumption of SCT [25,26] that individuals cognitively anticipate the likely consequences of their prospective behavior and more readily adopt behavior that is likely to produce desired outcomes rather than behavior that would bring undesirable or negative outcomes [76]. Three cognitive factors positively influence technology adoption (e.g., perceived benefits; self-efficacy; and perceived usefulness); whereas six cognitive factors negatively influence technology adoption (e.g., perceived risks; distrust; perceived ease of use; perceived cost; perceived threat to autonomy; and lack of interest).

##### 5.1.2.1 Perceived benefits

Perceived benefit of using technology is the most extensively identified cognitive factor influencing low-income older adults' technology use, emerging in 47% of studies (*n* = 8). The five facets of benefits are: health, social participation, self-actualization, mobility and monetary benefits. First, the most common benefit of technology adoption identified in the literature was perceived health benefits. For instance, low-income older adults perceived that: (i) adopting an IVA would support them with health tasks and in obtaining emergency assistance [64]; (ii) using a patient portal allows digital archiving and analysis; serves as a neutral communication medium; stores complete medical records; and offers the possibility of contextualized medical advice [70]; (iii) using telecare allows them to communicate with a clinician from home; calls for medical help when needed; helps manage medications [19]; and (iv) adopting Tele-BA would benefit them for online psychotherapy; and with obtaining the positive effects of increased physical activity on health and self-enjoyment [61]. Second, studies also found that perceived benefits in social participation encouraged older adults to use technology. For example, Kim and Gray [69] found that enhanced social connections through using the Internet experienced by low-income older adults encouraged their continued use; and Gallo et al. [68] reported that older adults' perception of electronic devices as enabling them to use technology-mediated social participation, such as using video chats to interact with people more personally; whereas Berridge [58] found that some older adults strategically ‘misused’ a remote monitoring system by transforming telecare calls into opportunities to chat with the operator to combat social isolation. Third, studies also showed that technology's benefits in fulfilling low-income older adults' self-actualization needs motivated their use of technology. For example, Kim and Gray [69] found that older adults use the Internet because it offers opportunities to develop their life skills. Similarly, Andonian [67] found that recognition of personal growth and change; contribution and engagement in relationships and the global community through computer use were the key factors driving older adults' computer use. Berridge [58] found that older adults adopted a remote monitoring system because they perceived the need to be ‘doing the right thing’ to reduce family burden. Another two aspects of perceived benefits – perceived monetary benefits and perceived mobility benefits – also influenced low-income older adults' technology use but the factors were each found in single studies and thus the conclusions that can be drawn are limited. For instance, studies found that low-income older adults used the Internet and electronic devices because they believed such use would benefit them financially – for example, making long distance calls using video calls or other Internet applications would save them monetary cost [69] and benefit them in managing their mobility [68].

##### 5.1.2.2 Perceived usefulness; and perceived ease of use

Cognitive evaluations of two technological aspects emerged from our analysis: perceived usefulness and perceived ease of use. Studies found that a high level of perceived usefulness influences low-income older adults to use technology; however, they were hindered by a low level of perceived ease of use. For instance, a high level of perceived usefulness motivated low-income older adults to use the Internet and computers, whereas a low level of perceived ease of use served as a barrier [65]. Usability issues due to system complexity was a barrier to patient portal use [70]; and reliance of the system on the user's ability to communicate with the operator impeded the adoption of a sensor-based remote monitoring system [58]. Nevertheless, studies examining technological evaluations were fairly limited. These factors were inductively derived in qualitative studies and are yet to be supported statistically in quantitative studies to determine the effect of technological beliefs on technology use.

##### 5.1.2.3 Self-efficacy

Self-efficacy emerged as another cognitive factor that influences technology use among low-income older adults. This is in line with the premise of SCT, that besides cognitive beliefs of expected outcomes, self-efficacy is the main cognitive force guiding an individual's behavior [25,26]. For instance, Gallo et al. [68] found that low-income older adults perceived their own (dis)ability in terms of physical ability and know-how as a barrier to using cell phones, smartphones or computing devices. Kim and Gray [69] found that older adults perceived their low literacy due to lower education attainment as a barrier to using the Internet, and their limited proficiency would limit the range of Internet use. However, quantitative findings on the effect of self-efficacy on technology use were inconclusive, probably because the effect was investigated in only two studies which respectively yielded both significant and non-significant effect. Jung et al. [62] found that computer self-efficacy was associated with enrolment in training and computer and Internet use, whereas [60] reported the association between self-efficacy and Internet and computer use to be non-significant.

##### 5.1.2.4 Perceived risks

The most commonly identified cognitive assessment negatively affecting low-income older adults' technology use was perceived risk, which emerged as a theme in four studies. Seo et al. [65] found that both privacy concerns and security concerns had caused low-income older adults to be hesitant to use the Internet and computer because they were ‘nervous’ about viruses and believed that technology would allow others to access their personal information. Similarly, Gallo et al. [68] reported perceived lack of safety by low-income older adults resulting in resistance towards electronic device use, because they had been “hacked already three times” on the computer. In the field of digital health technology, Latulipe et al. [70] reported that privacy concerns regarding personal medical records and security concerns for online systems were the main factors preventing low-income older adults' from using a patient portal. Likewise, confidentiality risks – concerns about privacy of personal health data – were a major concern about adopting an IVA [64]. These findings suggest that a lack of understanding and misconceptions about technology need to be addressed in order to increase low-income older adults' acceptance and use of technology.

##### 5.1.2.5 Distrust

Some studies depicted distrust as another factor influencing technology use among low-income older adults. For instance, Nallam et al. [64] reported low-income older adults who raised concerns about trusting the information provided by an IVA, such as whether up-to-date medical information was ensured when providing recommendations. Kim and Gray [69] found that suspicion or distrust of governmental or social programs was an obstacle for an Internet use intervention. These findings suggest that distrust towards technology and technological intervention programs negatively affects technology use among low-income older adults.

##### 5.1.2.6 Perceived cost

It is evidenced in studies that perceived cost has a negative impact on technology use among low-income older adults. For example, Gallo et al. [68] and Kim and Gray [69] reported low-income older adults’ concerns regarding the financial cost of using technology, such as purchasing smartphones, paying for technology-mediated services like ride-hailing, and the cost of broadband subscriptions, which were beyond their means, and this inhibited their use. Notably, in a resource-limited setting, monetary cost has an impact on technology use due to accessibility and affordability issues. Nonetheless, some studies showed that perceived cost which is non-monetary (e.g., anticipated loss) emerged as a factor affecting technology use. For instance, Latulipe et al. [70] found that low-income older adults responded negatively towards using a patient portal because the possibility of patient portal use replacing face-to-face visits by their health provider, which is perceived as a cost or loss. Few studies reported perceived cost explicitly as an emergent theme in their studies – perceived cost was described as interrelated with other factors of adoption, such as perceived benefits and accessibility of technology. For instance, older adults perceived the benefit of adopting an IVA because it would save the financial cost of an unnecessary trip to the doctor [64], whereas the perceived cost of technology was interrelated in one study with technology accessibility barriers [70]. These findings demonstrate that perceived cost is a salient factor in technology use among the low-income older adults, suggesting that addressing issues relevant to perceived cost is unavoidable in any technology adoption study or intervention for this segment of society.

##### 5.1.2.7 Perceived threat to autonomy

Another cognitive evaluation identified as a negative factor for technology use by low-income older adults is perceived threat to autonomy. For instance, in the study by Berridge [58], perceived threat to autonomy influenced older adults' use of a sensor-based remote monitoring system in their homes. Older adults discontinued the use of the system because they perceived that it was programmed with certain assumptions about how they would or should live, and hence posed a threat to their behavioral autonomy, particularly if their behaviors were irregular or did not conform to the “assumptions about ‘normal’ habitual behavior” and resulted in unnecessary alert signals. Some of those studied modified their behavior to avoid alert signals but eventually discontinued the use of the remote monitoring system. Others responded by ‘misusing’ the alert system to exercise control, such as by maneuvering on the floor in the event of a fall, to prevent alert signals being sent to family, so as to avoid hospital time.

#### 5.1.3 Affective responses (emotions)

SCT asserts that besides cognition, affect shapes behavior and that a dynamic interplay exists between thoughts, affects and actions [25]. Studies reported that negative affective responses (e.g., computer anxiety; and aging anxiety) are negatively associated with technology use among the low-income older adults, concurring with findings in general older adults’ technology adoption studies [77,78]. Nevertheless, inductively derived factors revealed that positive affective responses such as sense of freedom; interest; feeling productive and useful; hope; and pride are also significant predictors of technology use among the low-income older adults.

##### 5.1.3.1 Computer anxiety

Among the affective responses, the effect of computer anxiety emerged as the most significant factor negatively associated with technology use. For instance, computer stress and computer anxiety were found to be negatively associated with technology use [47,54). Kim and Gray [69] found that two types of computer anxiety – fear of technology due to the challenge it posed; and fear or misconceptions about cyber security – were obstacles that limited low-income older adults' Internet use. The anxiety induced by technology has also resulted in low-income older adults discontinuing its use, such as in the case of the remote monitoring system noted above. Berridge [58] reported that the older adults discontinued its use because they felt “uncomfortable, fearful and ‘spooked’” by the passive monitoring technology. These findings suggest that the feelings of fear, discomfort or negative emotional reactions induced through the thought of using technology or actual technology use would inhibit its use among low-income older adults.

##### 5.1.3.2 Aging anxiety and lack of interest

Aging anxiety was found to be negatively associated with technology use. For instance, Jung et al. [62] found that aging anxiety was negatively associated with low-income older adults’ enrolment in training and Internet and computer use, suggesting that older adults who felt anxious about change and the transitions that come with aging were less likely to adopt technology. Another barrier to technology use was the lack of interest observed in the study by Latulipe et al. [70], which found that low-income older adults lacked interest in technology use due to their perception of age and the absence of computing technology during their formative and working years.

##### 5.1.3.3 Freedom; feeling productive and useful; hope; pride and belonging

Studies have identified several positive affective responses as factors motivating low-income older adults' use of technology. For instance, freedom to express interests, values, and identity elicited from computer use have resulted in continued use of computers among low-income older adults [67]. Choi et al. [61] found that not only did older adults experience feelings of being productive and useful with the adoption of Tele-BA, but that the use of technology also elicited hope for the future with improved mood and social connectedness. In particular, older adults felt pride in doing tele-sessions and were motivated to learn technology. These positive affects influenced their decision to continue to use Tele-BA for their treatment. Belonging was another affective response that emerged in the literature; however, the effects of belonging on technology use were found non-significant. All three measures of belonging: feelings of belonging to a senior center; neighbourhood belonging; and feelings of social support were not significantly associated with enrolment and use of computers and the Internet [62]. Studies on positive affective responses related to technology use were relatively scarce, amounting to only 18% (*n* = 3). The paucity of literature was probably due to the limited range of technology types in the present low-income older adults’ technology adoption studies which focus primarily on the utilitarian aspects of ICT and digital health technology use with less focus on technologies and applications for hedonic outcomes. Although the number of studies were limited, these findings suggest that positive emotions towards a particular technology have a positive impact on the decision to use technology among low-income older adults.

### 5.2 Environmental factors

SCT posits that an individual's behavior is shaped and controlled by personal and environmental determinants [25]. In the present review, four environmental domains have emerged: social (i.e., social support/social capital); technological (i.e., accessibility of technology); physical (e.g., urban/rural, and housed/homeless); and organizational characteristics (i.e., insurance status). Environmental factors influencing technology use among low-income older adults are shown in Table 5. In general, environmental determinants have been studied less thoroughly in terms of breadth and depth in the existing literature; nonetheless, empirical evidence consistently showed that the social and technological environment are key environmental factors influencing low-income older adults' technology use.

**Table 5:** An overview of the environmental factors related to technology use among low-income older adults.

Dimensions of environmental determinant	Factor	Study
Social environment	Social support/Social capital	[[55], [56], [57],^60^]* [68]; (q)
Technological environment	Accessibility of technology +	[55,63]* [64,69,70]; (q)
Physical environment	Housed/homeless	[66]
	Urban/rural clinic	[57]*
Organizational characteristics	Insurance status	[57] n.s.

Note. *significant, n.s. nonsignificant, ^no correlation conducted, + aggregated and self-coded, (q) qualitative data (Refer Appendix B. Table B1 and Table B2 for details).

#### 5.2.1 Social environment

Based on the notion of SCT, human behavior is regulated by an interplay of self-generated and external sources of influence [46]. Personal behavioral change occurs within a network of social influences, including social norms and the behavior of people in one's immediate social environment, such as parents, friends, and other community members [25]. The present review found that low-income older adults' social environments (i.e., social support/social capital) is significantly associated with their use of technology, albeit the investigations were less thorough.

##### 5.2.1.1 Social support/social capital

In general, studies found that low-income older adults with social support are more likely to use technology. Social support was operationalized in terms of: marital status; living with a spouse; living with someone; having at least one living child; having at least one living sibling; number of people in the household structure; and having a care partner. Studies showed that the effect of marital status was significant across studies, indicating that low-income older adults who are currently married are more likely to use technology [55,57]. Older adults living with a spouse (as compared to those not living with a spouse) and having at least one living child (as compared to none) were more likely to adopt the Internet and computers [60]; whereas in contrast; having at least one living sibling (as compared to not having at least one living sibling) was found to be negatively associated with Internet and computer use [60]. Having support from family and friends encouraged older adults' use of ICT, especially with their children who had given them smartphones, computers, or tablets and taught them how to use them [68]. Further, social capital was found to be positively associated with technology use, showing the importance of individual resources from one's social network in facilitating Internet use [60]. However, contradicting result and non-significant effects were found in some studies. For instance, Choi & DiNitto [56] found that older adults living alone had a higher opportunity to use the Internet and computers; whereas Arcury et al. [55] reported the association between household structure (of one person as compared to two or three or more persons); and having a care partner and technology use to be non-significant. Nevertheless, it is evidenced in most of the studies that social support is significantly associated with technology use, despite the number of studies investigating social environment being limited.

#### 5.2.2 Technological environment

Investigating the effects of the technological environment is vital because technological factors are particularly relevant to information systems research. In the present review, technological environment refers to the technological aspects of an individual's environment that are available to support technology use; as postulated in SCT that environment and external sources in part influence individual behavior [25]. Although the number of studies investigating the effect of technological environment were relatively few, the present review found that technological environment (i.e., accessibility of technology) is a significant environmental domain influencing low-income older adults' use of technology.

##### 5.2.2.1 Accessibility of technology

Distinct from general older adults’ technology adoption studies, synthesis in the present review found that the accessibility of technology is a key factor affecting technology use among low-income older adults. Studies identified four aspects of accessibility of technology: technological devices; technological resources needed to use technology such as the Internet; technical support; and technology training. For instance, having two or more e-devices at home as compared to having one or none was positively associated with technology use [55]; whereas the lack of technology (e.g., computer) has prevented low-income older adults from using technology, such as patient portals [70]. Individual device needs affected technology adoption because technology was unaffordable for low-income older adults [69]. Studies found that the problem with maintaining technological resources needed to support technology use, such as the Internet connection using broadband services and wi-fi, was an access barrier to technology use because a subscription was unaffordable [64,69]. The lack of access to technical support hindered sustained technology use because technical assistance is imperative for low-income older adults who have lower computer and Internet proficiency [69]. Computer and Internet training not only impact technology use positively, but also increase technology use frequency [63]. Nonetheless, it is noteworthy that low-income older adults wanted to use technology because of its perceived benefits, but access to technology inhibited their use [64,69,70]. These findings suggest that addressing the issue of accessibility to technology is required at a higher level, such as through government policy and interventions, before technological initiatives are promoted at individual level.

#### 5.2.3 Physical environment and organizational characteristics

##### 5.2.3.1 Housed or homeless and care from urban or rural clinic

At present, there are relatively few studies investigating the influence of the physical environment and organizational characteristics on low-income older adults' technology adoption. Studies have found that physical environment (i.e., housed or homeless; and urban or rural clinic) are positively related to low-income older adults’ use of technology. For instance, older adults who have regained housing or who are housed rather than homeless are more likely to use the Internet [66], and older adults receiving care at urban rather than rural clinics are more likely to use a patient portal [57].

##### 5.2.3.2 Insurance status

The present review located only one study examining the effect of organizational characteristics (i.e., insurance status of having either private or government insurance versus none) on low-income older adults’ technology use. Nevertheless, the relationship was found non-significant [55].

### 5.3 Technology use behavior

Our systematic review and analysis found that most technology adoption studies examined the factors of actual use of technology, with only two studies investigating the intention to use (refer Appendix B. Table B1 & Table B2). Although studies have identified factors related to low-income older adults' technology use, there is no in-depth understanding of the usage behaviors. Several technology use-related behaviors emerged in this study, such as ‘intention to use’, ‘continued use’, ‘discontinued use’, and interestingly, ‘misuse’. In this review, the low-income older adults' usage patterns for each type of technology were identified and depicted in Table 6. Among four types of technologies used, predictably the broadest range of use was found in ICT, encompassing communication, information-seeking, management of various tasks and entertainment. Our findings suggest that the use of ICT among low-income older adults may be driven by various categories of needs from physiological needs to self-actualization needs. For instance, the usage of ICT for communication with social service agencies, shelters or housing providers and potential employers [66] indicates the important use of technology among low-income older adults to facilitate their access to physiological needs, such as food, a place to stay, and employment which are the basics for survival in a low-resource situation. ICT were also used to manage various tasks, such as paying bills or doing banking [56,60,65], shopping for groceries or personal items [56,60] and completing online paperwork [67] which is not only a necessity in a digital era but also minimizes the mobility challenges faced by older adults carrying out these tasks [79]. Similarly, the use of ICT for communication with medical provider [56,60,66], access to medical records [65], order or refill of prescriptions [60], obtaining health information [19,56,60,66], managing Medicare or other insurance matters [60] may be driven by safety needs to safeguard personal health and ensuring insurance protection, whereas the ICT usage for calling rideshare services [68], checking directions and bus schedules [68], and checking the news and weather [56,68] may be driven by safety needs in terms of mobility and traveling. ICT usage, such as call, message, video chat, read or send email to contact friends and family [56,60,[65], [66], [67], [68]], and check social networking or dating sites [56,66] could be driven by love and belonging needs for interpersonal relationships, social connection and intimacy. The usage of ICT to express one's values, such as Jewish identity, writing, and learning [67] could be driven by esteem needs for respect from others and elevating self-esteem. Usage patterns such as, learning [68], reading to enlarge one's sphere of knowledge or worldview [67], as well as the usage to express personal interests, such as virtual traveling and writing [67], video-viewing and listening to music [56,[66], [67], [68]], playing games [56,66] and browsing the Internet for fun [66] for the purpose of self-enjoyment suggest the uses of technology were for gratifying one's self-actualization needs.

**Table 6:** An overview of the type of technology, usage patterns, usage purposes and needs.

Type of technology	User		
	Patterns of usage	Usage purposes	Needs
ICT	• Contact social service agencies, shelters or other housing providers, potential employers, potential landlords • Look for information (about a shelter or place to live, job) • Pay bills or do banking • Shop for groceries or personal items • Complete paperwork online	Food Shelter Social services Employment Task-management	Physiological
	• Contact medical personnel • Look for medical information • Access medical records • Order or refill prescriptions • Manage Medicare or other health insurance matters • Call rideshare services • Check directions and bus schedules • Check the news and weather	Health Insurance Mobility	Safety
	• Communication-call, message, video chat, read or send email • Contact relatives and friends • Check social networking or dating sites	Interpersonal relationships Social connection Intimacy	Love and belonging
	• Express values (e.g., Jewish identity, writing and learning)	Self-esteem Respect	Esteem
	• Learning (e.g., the English language) • Read magazines or books • Enlarge sphere of knowledge/worldview • Express interests (e.g., virtual travel, writing) • Watch a video, download a music file, play games • Browse the Internet for fun	Self-enrichment Self-enjoyment	Self-actualization
Digital health technology	• View lab or test results • Send message or questions to doctor or nurse • Schedule or change an appointment • Manage medications • Refill prescriptions • Find health information • Call for medical help when needed • "Signal a nurse I'm OK" • "Monitor if/when I fall in my home" • "Detects if I'm able to get around as usual in my home" • Sends health information (e.g., blood glucose, blood pressure readings) to the doctor • Attend tele-sesseions, learn and practice behavioral activation (to improve self-care management of physical and mental health)	Health and well-being	Safety
	• Attend tele-sessions, learn and practice behavioral activation (to increase social connectedness)	Social connection	Love and belonging
	• Attend tele-sessions, learn and practice behavioral activation (to improve self-care management of physical and mental health, improve living environment and daily routine)	Self-worth	Esteem
	• Attend tele-sessions, learn and practice behavioral activation (to engage in self-enrichment and self-enjoyment activities)	Self-enrichment 'Self-enjoyment	Self-actualization
Smart sensor	• Passive use (e.g., reluctant use by obeying social workers at their subsidized independent living residential who represent a governing authority) and to make things easier for family	Independent living	Safety
	• ‘Misuse’ to combat social isolation (e.g., manipulate the technology to receive calls from the operators)	Social connection	Love and belonging
	• ‘Misuse’ to exercise control (e.g., manoeuvring to outsmart the system to avoid wasting time with unnecessary hospital care and troubling their emergency contacts)	Control Respect	Esteem
AI	• Assist with general decisions (e.g., recommending a medication) based on a verified fact • Research on illnesses or medications • Manage health • Obtain emergency assistance	Health Emergency assistance	Safety

Our systematic review also found the usage patterns of digital health technologies among low-income older adults were aimed towards health, social connection, self-worth, self-enrichment and self-enjoyment, suggesting the uses of technology were for the gratification of safety, love and belonging, esteem and self-actualization needs. For instance, patient portal was used to schedule or change appointment and refill prescriptions [57,70], find health information [57]; telecare was used to call for medical help when needed, signal a nurse a patient-user was OK, monitor if or when a patient-user fell in the home, detect if a patient-user is able to get around as usual in the home, send blood glucose and blood pressure readings to the doctor [19], whereas Tele-BA was utilized as online psychotherapy [61]. These usage patterns suggest that the use of digital health technologies could be a manifestation of fulfilling one's safety needs to protect one's health and well-being. The usage of Tele-BA for attending tele-sessions of psychotherapy to increase social connectedness with others may also suggest the motivation to satisfy one's love and belonging needs. Further, it was found that low-income older adult patients attended Tele-BA to learn and practice behavioral activation to improve self-care management of health and to improve their living environment and daily routine [61], suggesting the motivation of one's esteem needs to feel useful and to have a sense of self-worth. Through these tele-sessions, patients also engaged in self-enrichment and self-enjoyment activities, suggesting one's pursuit for self-actualization needs.

Another technology used by low-income older adults are smart sensor. Despite the function and purpose of this technology was to monitor the activity and movement within the living space to support aging in place and independent living, nonetheless, the findings observed unintended patterns of use, such as ‘misuse’ to combat social isolation and ‘misuse’ to exercise control. Interestingly, studies found that the need for social interaction was so pressing that some residents transformed telecare calls into opportunities to chat with the operator [58], suggesting the need for love and belonging acquired through having conversations with people who are concerned with their safety in the home. Besides, some residents manoeuvred to outsmart the system to avoid wasting time with unnecessary hospital care or troubling their emergency contacts [58], suggesting their esteem needs for a sense of autonomy and control of not wanting technology to dictate the outcome of their falls. Findings also showed that the smart sensor technology involved a passive use by the older adult user, who reluctantly accepted the technology to obey the social workers at the subsidized independent living residential with the goal of independent living, whereas some accepted remote monitoring to ease their family burden [59]. This usage patterns suggest the use of smart sensor may be driven by safety needs to secure their goal of independent living and for their family to feel safe when the older users were alone at home. One of the studies examined a more recent technologies, which is artificial intelligence (AI), in this case, an IVA. Our findings demonstrate the usage of an IVA among low-income older adults would be mainly for health, protection and information purposes, such as looking for information on illness or medications and obtaining emergency assistance [64], suggesting the motivation of using an IVA were to satisfy one's safety needs.

In summary, findings showed that ICT was used to facilitate access to physiological needs, such as food, shelter, social services, employment through communication with social service agencies, shelters or housing providers and potential employers. ICT was also utilized to minimize mobility challenges through managing various task online, such as bill-paying, online paperwork and grocery shopping. To fulfil safety needs, low-income older adults leveraged ICT and digital health technologies to safeguard their personal health in terms of obtaining medical help, prescriptions, information, and to preserve well-being through tele-sessions. Technologies were also used to attain a sense of security, such as ensuring insurance matters, ensuring safety for mobility and travel, obtaining emergency assistance, and to support safety for independent living through allowing remote monitoring. Love and belonging needs were gratified through the use of ICT to keep in touch with friends and family, visit social networking or dating sites and ‘misusing’ the operators' telecare calls from the remote monitoring system to have conversations. To fulfil esteem needs, it was found that low-income older adults used ICT for gaining respect from others, elevating self-esteem through expressing their values and identity, writing and learning online. Some used tele-sessions of psychotherapy to acquire a sense of usefulness and self-worth by improving their self-care management, their living environment and daily routine. We also observed that low-income older adults used ICT to attain self-actualization needs, such as self-enrichment and self-enjoyment by attending tele-sessions [61], enlarging their sphere of knowledge or worldview and expressing their personal interests online, through reading, learning, virtual traveling and writing [67,68], and self-enjoyment from video-viewing, listening to music, playing games and browsing the Internet for fun [56,65,66,68].

It is worth noting that among the seventeen studies, nine (53%) studies indicated the majority of the participants owned the technological devices they used, whereas the remaining did not own the technological devices and access to technology was made through loaned devices [61,69] and cybercafes in senior center [62]. Similarly, the studies of telecare [19] and IVAs [64] were both a priori acceptability wherein the low-income older adults have not afforded ownership or experienced the actual use of these technologies in their lives. Findings on technology ownership and usage of technology ascertained the important role of needs in motivating the use of technology among low-income older adults despite ownership. These trends of use and behavioral intention to use technology imply that in order to fulfil specific needs which they believed could be attained through the use of technology, this segment would find means to use technology (e.g., ICT) and were open to technological interventions (e.g., tele-psychotherapy, telecare, IVA) despite not owning technology. The details of technology ownership and usage patterns for each type of technology is shown in Appendix C. Table C1.

## 6 Discussion

The present literature represents a commendable effort by researchers in examining factors influencing low-income older adults' technology use which is vital to encourage technology use among this sector; informing policy and eventually supporting the implementation of technology-mediated solutions for aging in the low-income setting. Nevertheless, in the absence of a comprehensive and integrative theoretical framework, it is difficult to understand why low-income older adults adopt or resist technology and thus how best to provide leverage points for intervention. This review synthesizes the factors into a SCT-based framework [3] and further integrates Maslow's hierarchy of human needs [27,28] to elucidate technology adoption in the present low-income context.

### 6.1 Personal factors related to technology use

Empirical evidence suggests that in a low-income older adults’ context, both personal and environmental factors (i.e., cognitions, affects, sociodemographic characteristics; technological and social environment) are significant predictors of technology use. Within the personal determinant concerning the sociodemographic factors, age, ethnicity, educational attainment and level of income were the most significant predictors, whereas other sociodemographic factors were either less studied or yielded mixed findings. In general, our finding is congruent with a recent machine learning study [80] which argues that socioeconomic variables (i.e., measures of education and income) will continue to predict technology use in aging in the future because financial standing is difficult to address, yet socioeconomic resources are pre-requisites to basic technology use. Nevertheless, our findings of the patterns of technology use and technology ownership suggest that low-income older adults were willing to use technology if the usage could address their needs and if technology were provided to them. Our assertion is supported by a prior study [81] which found that income appeared to be a modifying factor, but not a main factor driving patient portal adoption among disadvantaged populations. Therefore, given the present socioeconomic context, the present review suggests that in order to address the digital divide among this segment, strategic policy and intervention (e.g., subsidized or special technology packages) is imperative so that low-income older adults can utilize and benefit from technology use despite their socioeconomic status.

The findings on gender concur with prior systematic reviews of general older adults' technology adoption which found the effect either inconsistent or non-significant [3,5,21]. The findings on gender, language and employment suggest that not all sociodemographic variables are consistent and salient factors for predicting low-income older adults' technology use. However, sociodemographic and cultural context might shape individuals' beliefs driving technology adoption behavior. In summary, the present study suggests that individuals' beliefs may be more robust predictors of low-income older adults' technology use. This leads to the first research question suggested for future studies (RQ1): *How do sociodemographic and cultural context influence low-income older adults’ beliefs driving their technology adoption behavior?*

As detailed in the preceding section, we identified that individuals' cognitive evaluations and affective responses emerged as the key domains influencing low-income older adults' technology use. Among these domains, perceived benefits emerged as the most prominent factor in driving technology use. Another similar factor emerged was perceived usefulness which also motivated the use of technology. Despite empirical evidence showing that both perceived benefits and perceived usefulness influence low-income older adults' technology use, these concepts are closely associated and the distinction between what constitutes perceived benefit and perceived usefulness was unclear in the extant literature. These factors were mainly derived inductively from qualitative studies without distinct operationalization of the constructs, thus (i) more research is needed to examine the role and effects of perceived benefits and perceived usefulness on low-income older adults' technology adoption, and (ii) any differences between these constructs driving technology adoption behavior need to be delineated. This leads to our second and third future research questions: (RQ2) *How do perceived benefits and perceived usefulness influence low-income older adults’ technology adoption?* and (RQ3) *How can we distinguish perceived benefits and perceived usefulness by delineating the differences between them?*

The perceptions of costs in these studies suggest a multidimensional characteristic of costs consists of financial cost, such as purchasing a technological device and subscribing to a broadband service [68,69] and cost in terms of a loss or sacrifice, such as patient portal replacing face-to-face visits by health provider [70]. Besides the cost of adopting technology, studies in our review also indicate a notion of the potential costs of not adopting technology, such as unnecessary trip to the doctor [64], suggesting the consideration of effort and probably financial cost related to non-adoption of technology. These findings are consistent with existing older adults’ technology adoption studies [21,23]. Given its pertinent role, a deeper understanding of what constitutes perceived cost and its role and effect is required to provide practical insights. This leads us to the fourth future research question: (RQ4) *How do the different costs of adopting or not adopting technology influence technology adoption among low-income older adults?*

Cognitive evaluations, such as perceived ease of use, self-efficacy, perceived risk, distrust and perceived threat to autonomy were also identified to negatively impact technology use among low-income older adults. Specifically, the finding of perceived threat to autonomy is congruent with general older adults' studies which have identified that fulfilling the need for autonomy is important to encourage technology adoption [82], suggesting that preserving autonomy is pivotal when developing technology for older adults, whatever their background. Affective responses with a negative connotation, such as computer anxiety, aging anxiety and lack of interest were found to inhibit low-income older adults' use of technology, whereas positive affect of freedom, feeling productive and useful, hope, pride and belonging have a positive impact on the decision to use technology. In general, these findings are congruent with the findings from systematic reviews of technology adoption among older adults in general [3,4,23], suggesting that factors of personal cognitions and affects are pivotal for guiding technology design and development, and for intervention at personal level. Empirical evidence in the present review consistently showed that the psychological process of cognitions and affects were essential factors relating to low-income older adults' technology use. However, the number of studies were limited, implying that more studies are needed to obtain in-depth understanding in order to draw meaningful conclusions. Furthermore, the use of a theoretical foundation to conceptualize cognitive and affective factors influencing technology adoption was missing in the low-income older adults' literature in spite of the emphasis on theory-driven research in behavioral studies. Based on this discussion, (i) more research is needed to fully understand the antecedents and underlying mechanisms of the cognitive and affective process driving low-income older adults' use of technology, and (ii) theory-based conceptualization needs to be incorporated. This leads to our fifth and sixth suggestions for future research: (RQ5) *What are the antecedents of the cognitive evaluations and affective responses driving low-income older adults' technology adoption and the underlying mechanisms?* and (RQ6) *How can existing theories and frameworks be adopted to explain low-income older adults’ technology adoption behavior?*

### 6.2 Environmental factors related to technology use

Within the environment determinant, empirical evidence in the extant literature found that the social and technological environment has an impact on low-income older adults' technology use. Studies showed that social support, such as marital status [55,57], living with a spouse [60], living alone [56], having at least one living child and having at least one living sibling [60], having support from family and friends [68] and social capital [60] were found to be significantly related to technology use. Notwithstanding the evidence, it remains unknown how and to what extent social environment influences low-income older adults' use of technology because the present literature focused mainly on the properties (i.e., social support) of social environment, but investigations on the effect of social influence on technology use were missing in comparison to the findings in prior systematic reviews [5,21,23]. In terms of technological environment, the lack of accessibility to technology [55,70] was a key factor inhibiting its use – low-income older adults generally lack technological devices and the technical resources needed to enable their use. Notably, accessibility to technology was rarely reported as a main factor in recent systematic reviews on general older adults' technology adoption, instead it was briefly accounted for in the contruct, “facilitating conditions” [43,77], suggesting that this factor might be more prominent among low-income older adults. Specifically, it was voiced by low-income older adults that technology was unaffordable [64,69]. This finding suggests that, with limited disposable income, addressing imminent needs, such as physiological needs for food and shelter would be prioritized among this segment, and they would be more likely to use technology if it was made free and available to them. Given that accessibility and affordability are pre-requisites to technology use, strategic interventions at government and organizational levels are thus required. Nevertheless, understanding of the reciprocal effects between these environmental factors and low-income older adults' cognitive evaluations and the affective responses driving their technology use behavior could provide contextual understanding and valuable insights for interventions at individual level. This leads us to the seventh and eighth future research proposal: (RQ7) *How does the social and technological environment influence low-income older adults' cognitive evaluations and the affective responses driving their technology adoption behavior?* and (RQ8) *How does low-income older adults’ cognitive evaluations and the affective responses affect social and technological factors in shaping their technology adoption behavior?*

### 6.3 Technology use behavior and embeddedness of fundamental human needs

Although needs related to technology use was not explicitly examined in these studies and was only briefly described in some studies [56,64], nonetheless the patterns of usage behavior showed that low-income older adults utilized technologies to reach their fundamental human needs encompassing physiological, safety, love and belonging, esteem and self-actualization. First, our analysis of technology usage patterns in these studies indicates that low-income older adults used technology for needs gratification, suggesting the implicit role of needs underlies technology use among this segment. Our findings are consistent with prior systematic review [4] which found that the need for technology influences technology use among older adults. The effect of needs is also observed in a recent study which found that belonging need is closely related to intention to use technologies and services [83]. In this review, technology use behavior observed in the usage patterns shed light on the use of technology to gratify fundamental human needs, suggesting these needs are the background factors underlying the use of technology among low-income older adults. Hence, the development and successful adoption of technologies for this segment will depend, among other personal and environmental factors, on analyzing, planning, responding to the needs of potential older adult users. Despite how powerful, unobtrusive, inexpensive, or intelligent a technology is, older adults will be unlikely to adopt it if it does not fulfil their relevant needs [84]. Therefore, given the embeddedness of fundamental human needs which plays a central role underlying technology use among this segment, we contend that needs are not merely one of the factors related to technology use as indicated in prior systematic review [4], instead needs are embedded and represent the anchoring background factors exerting influence on technology use. Therefore, in our attempt to provide a comprehensive understanding, our framework outlined the person and environment determinants and highlighted the embeddedness of fundamental human needs which plays a central role underlying technology use among low-income older adults.

Second, the analysis of patterns of use also revealed that various categories of needs coexist and are fulfilled at the same time through technology use, suggesting that needs fulfilment are not necessarily sequential following the hierarchy, instead various layers of need could be fulfilled simultaneously through technology use. Our assertion is supported by a recent empirical study by Ref. [73] which found that the satisfaction of physiological needs is not a precondition for people to be adequately satisfied in higher layer needs. Therefore, policymakers and technology developers should not sideline but instead endeavor to expand the capacity of technological interventions for the satisfaction of needs as important as love, belonging, and esteem [73] in developing technologies that support aging for this segment.

### 6.4 Low-income older adults and prioritization of needs in technology use

In general, this review and synthesis suggest that specific personal and environmental factors are salient among the low-income segment, particularly factors regarding income, accessibility to technology and perceived cost are predominant in a resource-limited setting, suggesting the notion of technology use being unaffordable. The types of technology used also depicted the use of technologies consist of the most ubiquitous technology (e.g., Internet and computer, mobile technology) and were mainly technologies focusing on functionality and utility purposes (e.g., patient portal, telemedicine and smart sensor) instead of technologies for hedonic purposes. The narrower types of technology used and their technology usage patterns jointly affirm their prioritization of technology use were to address embedded fundamental human needs depended on affordability. This is supported by a recent observation of smartphone adoption at the bottom-of-pyramid by Baishya and Samalia [10] who found that both performance expectancy and perceived monetary value showed stronger impact for older people compared to younger people, suggesting that older people of low-income are forced to emphasize utilitarian needs as compared to hedonic needs due to the increasing family responsibility with the limited disposable income. Researchers have argued that “as a minimum, humanitarian needs assessment should consider actual or imminent threats to life, health, subsistence and physical security (protection)” [85]. Taken together, it is reasonable to suggest that the prioritization of fundamental needs to sustain their daily living were also reflected in their cognitive prioritization of needs in technology use. Notably, in a precarious context facing limited resource on top of pressing needs, if there are necessities and fundamental needs that technologies can provide and a free access is made available, it is more likely that low-income older adults would adopt technology. This trend is in contrast with technology use among general older adults [86,87] because it is unlikely that this segment would make financial sacrifices to accommodate the use of technology merely for hedonic purposes. Therefore, we contend that the layers of hierarchy are relevant to understand the prioritization of needs in technology use in the present context concerning technology adoption among low-income older adults. The representation of hierarchy of needs in technology use in our integrative framework illustrates that there would be greater motivation to use technologies that facilitate the fulfilment of the lower layers of need, namely physiological needs, followed by safety needs, love and belonging and the subsequent needs. It is important to reemphasize that the proclivity of needs in technology use does not deny the assumption that various levels of need coexist and does not contradict with the capacity of technologies in fulfilling multiple layers of needs simultaneously [73].

Nevertheless, there is no in-depth understanding of the interrelations between technology use-related behaviors, usage patterns, the role of embedded fundamental human needs and personal or environmental factors influencing technology use. For instance, the patterns of usage indicated the embeddedness of fundamental human needs, however, the effect of needs were not explicitly investigated in these studies, suggesting a possible confounding effect of needs in the technology adoption. Given that it is unclear how these dimensions are connected and interrelated, the present review proposes the ninth and tenth research question: (RQ9) *How are different factors and needs related to different technology usage behaviors among low-income older adults?* and (RQ10) *How can technology usage behaviors impact the lives of low-income older adults which influence continued use of technology?*

### 6.5 Future research avenues

Based on our observations and discussion in the preceding section, we discuss the research gaps and opportunities and propose avenues for future research. In this section, we organize questions identified into three future research areas and discuss potential collaboration across disciplines. Based on the 10 research questions for future studies proposed in the previous sections, Table 7 presents an overview of the avenues for future research and related research questions.

**Table 7:** An overview of future research opportunities and research questions.

Research avenues		Research questions
1	Roles and effects of sociodemographic and cultural context, and reciprocal effects between social and technological factors and low-income older adults' cognitive evaluations (i.e., beliefs) and affective responses (i.e., emotions) driving technology use behavior.	RQ1 *How do sociodemographic and cultural context influence low-income older adults' beliefs driving their technology adoption behavior?* RQ7 *How does the social and technological environment influence low-income older adults' cognitive evaluations and the affective responses driving their technology adoption behavior?* RQ8 *How does low-income older adults' cognitive evaluations and the affective responses affect social and technological factors in shaping their technology adoption behavior?*
2	Psychological process and applicability of theories and frameworks.	RQ4 *How do the different costs of adopting or not adopting technology influence technology adoption among low-income older adults?* RQ5 *What are the antecedents of the cognitive evaluations and affective responses driving low-income older adults' technology adoption and the underlying mechanisms?* RQ6 *How can existing theories and frameworks be adopted to explain low-income older adults' technology adoption behavior?* RQ2 *How do perceived benefits and perceived usefulness influence low-income older adults' technology adoption?* RQ3 *How can we distinguish perceived benefits and perceived usefulness by delineating the differences between them?*
3	Behavioral patterns, needs and effects of technology use.	RQ9 *How are different factors and needs related to different technology usage behaviors among low-income older adults?* RQ10 *How can technology usage behaviors impact the lives of low-income older adults which influence continued use of technology?*

#### 6.5.1 Research avenue 1: roles and effects of sociodemographic and cultural context, and reciprocal effects between social and technological factors and low-income older adults’ cognitive evaluations (i.e., beliefs) and affective responses (i.e., emotions) driving technology use behavior

Observing the extant literature through the lens of SCT revealed the potential avenues for future research. As illustrated in Fig. 4, some directions of the relationships postulated in SCT were not covered in the studies reviewed. The first potential research opportunity is to study the interaction between environment and person leading to technology use behavior in line with the proposition of SCT that an individual's thoughts and behaviors are shaped by that individual's environment and vise versa [25]. First, future research can examine the roles and effects of sociodemographic and cultural context on low-income older adults' cognitive evaluations (i.e., beliefs) and affective responses (i.e., emotions) driving their technology adoption behavior. From the literature, we note that specific sociodemographic variables have statistically proven significant associations with technology use among low-income older adults. However, to determine what distinguishes this segment's adoption behaviors requires deeper contextual investigations to gain understanding of the beliefs driving their adoption behavior, shaped by their sociodemographic and cultural context, at both micro and macro levels. For example, at the micro level, how does personal or micro sociodemographic and cultural context influence an individual's beliefs driving technology use behavior? At the macro level, how does the macro sociodemographic, such as the economic and cultural setting in a middle-income or low-income country affects beliefs driving technology adoption behavior? Future studies could also examine how technology adoption factors vary across different macro settings (e.g., country and region).

Further, study of social and technological variables could provide insights for future interventions of technology-mediated solutions for this segment of society. Future studies could examine the reciprocal relationship between environment (e.g., social and technological variables) and personal factors (e.g., cognitive evaluations and affective responses) shaping technology use behavior. For instance, in-depth understanding of the interaction between social environment (e.g., social influence) on one's cognitive evaluations (e.g., self-efficacy, perceived benefits, perceived risks, etc.) shaping technology use could help practitioners in leveraging social platforms, such as older adult activity centres, to encourage technology use among low-income older adults. Similarly, interaction between one's cognitive evaluation (e.g., perceived costs, self-efficacy, etc.) or affective responses (e.g., computer anxiety, aging anxiety, etc.) and technological environment (e.g., training program, subsidized package, etc.) influencing technology use behavior can provide potential ways for interventions. Moreover, a better understanding of the influence of the technological environment could support policy and interventions to provide sustainable technological accessibility for low-income older adults, such as free Internet and technology packages for this segment of society, perhaps through a public-private partnership to address the accessibility issue of technology ownership as a pre-requisite to technology use.

#### 6.5.2 Research avenue 2: psychological process and applicability of theories and frameworks

The second future research avenue is focusing on a comprehensive understanding of the cognitive evaluations and affective responses of low-income older adults' technology adoption and how we can draw upon existing theories to explain the psychological process influencing adoption behavior. The previously discussed studies have provided empirical evidence to support the influence of cognitive evaluations (e.g., perceived benefits, self-efficacy, perceived cost) and affective responses (e.g., computer anxiety, freedom, pride) on technology adoption. However, little attention has been devoted to understanding what influences these cognitions and affects. For instance, studies have identified perceived cost as a salient factor for technology use among low-income older adults; however, little is known about the role and effects of different perceived costs, either monetary or non-monetary, of adopting and not adopting technology. Hence, it will be valuable for future researches to rigorously examine cognitive evaluations and affective responses, by exploring their antecedents and the underlying mechanisms driving technology use behavior among this segment. Cognitive predictors have been studied extensively in IS literature; however, studies examining the role of affects in IT use remain relatively few [88]. Affective responses are emotions felt towards technology, consisting of positive and negative emotions [88,89]. In particular, prior research has called for more explicit attention on the role of emotions in IT use [90,91]. For instance, social scientist researchers have found that generally, older adults’ future time perspectives and subjective age, influence their beliefs regarding what is valuable to them emotionally, shaping their decision-making in selecting the activities they would prefer to engage in Ref. [92]. Therefore, in the context of low-income older adults, it is imperative to understand the antecedents leading to beliefs, emotions and the underlying mechanisms that are germane to their technology adoption behavior.

To date, few investigations have focused on the perceptions of the technological aspects which impact technology use among low-income older adults. Although we found a plethora of IS studies examining the effect of cognitive evaluation of technological performance (i.e., perceived usefulness and perceived ease of use) on older adults' technology adoption [2], it was surprising that in the low-income literature, technological characteristics and how they affect technology use was less thoroughly investigated, thus suggesting a disciplinary gap in this area of research. Investigating the effects of cognitive evaluations of technological performance is vital because they are particularly relevant to IS research and imperative to enable prescriptions for actionable interventions. These constructs need to be expanded and adapted to new contexts. For instance, does TAM remain valid to explain technology adoption in a resource-limited context? In particular, to ensure the implementation fidelity of technology-mediated solutions to support aging needs among the low-income population, clear guidance in technology design is imperative. For example, thoughtful technology design needs to consider low-income older adults’ technological compatibility, accessibility and competence in technological skills. Technology development needs to prioritize ease of use, such as practical and simplified features, including the use of appropriate graphics to cater for low literacy, and low physical and cognitive abilities. Realistically, one-size-fits-all design for a broad older adult population is unlikely to be effective, given the different context of low-income older adults facing a range of practical challenges. Further, the present literature uses similar descriptions for perceived benefits and perceived usefulness of technology without any clear delineation of the difference between these constructs. Therefore, a clearer and more scrutinized concept of perceived benefits and perceived usefulness would provide researchers with deeper understanding and clarity in future research.

The present review also demonstrates the lack of theoretical foundation in conceptualization of studies in the extant literature. Most of the published studies provided only descriptive discussions of the relevant factors, with relatively few that drew upon existing theory to explain technology use behavior. For example, a few studies considered theory and existing models, such as TAM, in their studies. However, theoretical investigations were limited because researchers only reported participants’ evaluations of the constructs from TAM (i.e., perceived usefulness and perceived ease of use), but did not conduct correlation analysis between these constructs and technology use, hence limiting the explanatory purpose of the model applied. In a review study on eHealth literacy intervention [93], the researchers argued that existing interventions are not theory-based, and thus impede successful interventions. Future studies should therefore refrain from piecemeal approaches in the conceptualization of studies, instead considering theoretical foundations in research that provide a systematic understanding and valuable implications for practice.

#### 6.5.3 Research avenue 3: behavioral patterns, needs and effects of technology use

The third future research avenue is providing a more comprehensive understanding of how different factors are related to technology use behavior by delving deeper into the behavioral patterns and the role of embedded fundamental human needs in technology use. The present review showed that needs were reflected in technology usage patterns, however, were not explicitly investigated, suggesting a possible confounding effect of needs in low-income older adults’ technology adoption studies. The role of specific needs can be examined to understand whether needs influence the effect of personal cognitive evaluations, affective responses or environmental factors on the use of technology. Similarly, qualitative approaches would greatly enrich our understanding in this area by elucidating the layers of factors and reveal how these factors are interrelated to provide valuable insights of the phenomenon [94]. Further, future research can endeavor to examine technology adoption by employing the perspective of fundamental needs. For instance, researchers can examine factor influencing the use of technology from the perspective of safety needs reflected in a protective behavior [95], or understanding technology adoption from esteem needs such as, how esteem needs were related to resistance towards certain technologies. This is in line with the call by scholars who have advocated for a diversified perspective to re-examine how perceptions and expectations relate to technology adoption behavior [96] further enrich IS adoption research. The present review showed that little is known regarding the various behavioral patterns of technology use among low-income older adults, and more research is needed to understand fully which factors were related to different usage behaviors. Notably, successful implementation and meaningful use of technology is the preeminent goal to support independent living [6] and social participation [7,8] in this segment and, thus, practical insights are needed to achieve this goal. For instance, future research can identify the full range of behavioral patterns, such as intention to use, initial use, continued use including usage level, misuse, discontinued use, resistance, and so on, to establish which beliefs drive different behavioral patterns. This would not only reflect more accurately the various facets of adoption behavior but could also provide a more systematic and comprehensive framework to guide future researchers and interventions.

As illustrated in Fig. 4, the impact of technology use on the person and environment is not covered in the studies reviewed. Examining this aspect of the reciprocal relationship is imperative to understand how the effects of use influence the continuous usage of technology. Thus, study of the effects of technology use on the lives of low-income older adults and their aging and social environment is essential and should be prioritized in future research for two reasons: (i) evaluating the impact of technology adoption is pertinent for evidence-based and effective interventions; and (ii) to provide a holistic understanding of technology adoption phenomena, based on the theoretical propositions of SCT that person, environment and behavior influence each other in a triadic reciprocal manner [45]. For instance, researchers can focus on the outcomes of technology adoption on the person (e.g., self-efficacy, health knowledge and attitude, independent living, social participation, life satisfaction and quality of life) and the environment (e.g., social capital, social support, indoor and outdoor age-friendly environment) of low-income older adults.

### 6.6 Advancing the ‘low-income older adults’ research agenda: the way forward

Technology for older adults encompasses a wide range of devices and applications [5,21,23]. However, much of the literature on low-income older adults' technology use was focused primarily on the Internet and computer use, and a few studies on digital health. This lack of diversity of technologies in the literature reflected the limited research attention to this segment of society. Most research into low-income older adults' technology adoption originated from the fields of gerontology, aging, medical informatics and healthcare, wherein the stakeholders' aims were to introduce technology-mediated solutions (e.g., telecare, tele-BA, patient portal, etc.) to support aging among this segment and reduce disparities in aging and health, reduce healthcare and insurance costs, institutionalization, and the need for social workers. In the face of a demanding economic situation and the rapid increase in old age dependency in an aging world, especially in the developing countries (WHO, 2020), the need for relevant stakeholders to implement technology-mediated solutions to support aging needs among this segment is anticipated to increase rapidly. Nevertheless, the effort to harness resources through technological platforms would be in vain unless older adults adopted the technology and benefitted from its meaningful use [58]. Therefore, future low-income older adults' technology adoption studies need to consider collaborations across disciplinary borders to harness integrated insights that enrich not only the IS literature but also the aging and low-income literatures. More IS researchers are needed to become involved, to advance this research agenda. Theories and frameworks need to be expanded and adapted to new contexts to derive a contextualized understanding of low-income older adults' technology adoption behavior. For instance, how can TAM and its extensions remain robust as a means to explain technology adoption in a resource-limited context? How can psychological and biological variables (e.g., cognitive evaluations and cognitive function) and environmental factors be integrated to better explain technology use behavior? Furthermore, technology adoption in this segment of society is complex and subject to multi-faceted influences. Given that varied research foci across disciplines may result in fragmented knowledge, the present review argues that this can be reconciled with a paradigm shift, by leveraging different typologies of theory to understand and explain low-income older adults’ use of technology. Following this approach, researchers can move beyond the mainstream narrower approach of testing existing theoretical models which impede multidimensional understandings of aspects of reality in a complex phenomenon [94,97]. Instead, research can draw together interdisciplinary knowledge to provide more informed and inclusive insights for both research and practice.

## 7 Contributions

Using a systematic approach, the present review has identified the trends, disciplines, theoretical foundations and factors influencing low-income older adults' technology use in the prior studies, and proposed avenues for future research based on the gaps identified in the extant literature. This review study contributes to both research and practice. First, this study provides a consolidated understanding of the factors influencing older adults' technology adoption, distinct from prior systematic review studies [4,5,21,23,80] in terms of: (i) specific representation of low-income older adults rather than older adults from a broad and general population, and identified factors salient among the low-income segment; (ii) mapping of determinants, domains and factors using a SCT-based framework provide an overview of factors observed and opportunities for future investigations; and (iii) incorporation of Maslow's hierarchy of needs into the SCT-based framework enhanced theoretical understanding on technology adoption in specific contexts [44]. Such an integration suggests some important additions to and expansions of SCT's model of human behavior with the notion of a hierarchy of fundamental needs embedded in human's behavior (as illustrated in Fig. 4), which was missing in prior SCT-based older adults' technology adoption framework [3]. Anchoring our framework upon these two theories enables a holistic perspective of how interactions between various determinants (e.g., cognitive and affective evaluations, environment, and human motivational systems of fundamental needs) can be examined to understand and promote technology use among low-income older adults. Further, although Maslow's theory faced criticisms by analysts who suggested that Maslow sometimes lumped together distinct needs into single, overly broad categories [74], however, the analysis in this study supported the broad-based view of Maslow's theory which is relevant for understanding technology use in a precarious context concerning the low-income older adults. Specifically, the embeddedness of a hierarchy of fundamental needs is observed to underlie technology use behavior in the present context. This study also provides support to the latest development of Maslow's theory that needs are prioritized, but are not necessarily met sequentially, consistent with the latest empirical evidence [73]. With this review, researchers can obtain an overview of literature across disciplines investigating factors influencing low-income older adults' use of technology. Future studies can extend the proposed integrative framework by examining the reciprocal relationships not discussed in the present study (as illustrated in Fig. 4). Discussions of future research avenues should help future studies to develop research foci that advance the low-income older adults' technology adoption literature.

Second, the present review has identified several limitations of the existing literature which have implications for future research: (i) the lack of investigations anchored on theoretical foundation; (ii) inadequate depth and breadth of current literature and the resulting inability to draw conclusions regarding the effects of specific factors; and (iii) the disciplinary gap in the literature wherein different research foci resulted in fragmented understandings of the phenomena. To our best knowledge, this study is the first to review systematically and provide an integrated interdisciplinary perspective on this topic of interest. In general, this review is expected to draw research attention across multiple disciplines, especially in IS, and to encourage researchers to venture deeper into low-income older adults’ technology adoption research to better understand contextual effects on psychological processes driving technology use. This study suggests that IS researchers have a pertinent role to contribute to both research and practice: (i) to enrich existing literature by incorporating theoretical investigations and reconciling disciplinary knowledge gaps; and (ii) providing useful insights for successful implementation of technological interventions to support aging for the low-income segment.

Third, this systematic review has several implications for policy and practice: (i) it provides insights for various stakeholders, such as governments, IS developers and aged care service providers seeking to leverage technology-mediated solutions for sustainable aging among low-income older adults; and (ii) it enables IS managers in the aged care industry and social work to better understand the motivation of embedded fundamental needs, benefits and barriers that users perceive and experience regarding use of technologies, allowing them to take practical and relevant measures and provide support to encourage low-income older adults to use these technologies. For instance, technological development and intervention should consider explicitly how a technology can satisfy particular levels of need and to promote uptake by advocating how technology use would be effective in addressing relevant needs. These are important contributions because without systematic synthesis of a wide spectrum of factors and the salient contextual background of fundamental needs, it is difficult for managers to prioritize particular measures and effectively address needs through technological interventions. Specifically, our study informs various stakeholders of the positive factors that we can magnify through technological interventions, and identifies the negative factors that we should minimize, such as by addressing issues pertaining to accessibility of technology, correcting misconceptions on risk, privacy and security through technology awareness programs; and reducing computer anxiety and increasing perceived ease of use through recurrent training.

## 8 Limitations

The first limitation of the present review is the language for literature search which included only studies published in English, thus may have missed studies published in other languages, for example there was no representation of studies conducted in non-English speaking regions. The second limitation is the possibly limited scope of terms used in our systematic search for literature – the terms for types of technology were not exhaustive. Third, the pool of technology adoption studies is relatively limited because literature with a low-income older adults’ focus is at the emerging stage, and thus the factors presented are less than thorough and merely reflect the status of the current literature. More research is needed to enrich the present framework, especially the relationships that have not been observed in prior literature in order to provide a holistic perspective to understand the interaction between person, environment and behavior determinant shaping technology use among low-income older adults. Fourth, all of the studies reviewed were conducted in the USA, a developed nation with generally higher digital literacy and digital technology usage rates than developing countries. Thus, the discussions were primarily centered on this population and might not accurately represent low-income older adults in different economic and cultural settings. Consequently, the findings face limitations of generalizability and future research is required to validate the results among a broader population of diverse economic and cultural settings, such as in a developing country in a different geographical region.

## 9 Conclusion

In summary, findings from the present review suggest that both personal and environmental factors, such as cognitions, affects, sociodemographic characteristics, technological and social environment are significant predictors of technology use in a low-income older adult context. However, specific factors are more salient among the low-income segment, mainly income, accessibility to technology and perceived cost. More importantly, the technology usage behavior shed light on the embeddedness of fundamental human needs which plays a central role underlying technology use among low-income older adults. On the basis of the existing evidence, addressing these pre-requisites as well as the gratification of fundamental needs is pivotal to ensure that technological design, development and interventions can yield effective implementation. However, further research is needed to obtain holistic and in-depth understanding of the interplay between person, environment and behavior determinant shaping technology use among low-income older adults from diverse economic and cultural setting. More research is needed to shed light on the antecedents and underlying mechanisms of psychological processes, anchored upon theoretical foundations. In particular, research collaborations across disciplines are imperative to reconcile the disciplinary knowledge gap and provide useful insights for actionable interventions.

## Credit author statement

**DYLC**: Conceptualization; Conduct of systematic review; Data analysis; Writing – original draft. **SWHL**: Conceptualization; Conduct of systematic review; Methodology; Project administration; Supervision; Writing-review and editing. **PLT**: Conceptualization; Conduct of systematic review; Methodology; Funding acquisition; Project administration; Supervision; Writing-review and editing.

Declaration of competing interest and funding source.

The authors declare that they have no known competing financial interests or personal relationships that could have appeared to influence the work reported in this paper.

## Data availability

No data was used for the research described in the article.

## Additional information

No additional information is available for this paper.

## Declaration of competing interest

The authors declare that they have no known competing financial interests or personal relationships that could have appeared to influence the work reported in this paper.

## Figures and Tables

**Table A.1:** Empirical studies on factors influencing technology use among low-income older adults (*n=* 17)

Reference	Technology & purpose	Country	Sample size	Study design/Analysis	Theory/Model	Factors/Themes	Behavior
Raven et al., 2018	The Internet and mobile technology - communication, search for information, health and non-health related task	USA	n = 277 Note: total 300 sample, but analysis is done with 277 who are users	Researcher administered survey/Multivariate regression analysis	None	Housed/homeless; health condition/history; ethnicity	Use (Internet and mobile technology)
Arcury et al., 2017	Patient portal - to improve communication with providers; access health information that improves decision making	USA	n = 200	Researcher administered survey/Multivariate logistic regression analysis	TAM and Person-Environ-mental Interaction Model	Ethnicity; education; social support; urban/rural clinic; experience; health condition/history; insurance status	Use (Patient portal)
Choi & DiNitto, 2013b	Internet and computer - communication, search for information, health and non-health related task	USA	n = 756	Researcher administered survey (Face-to-face or telephone)/multinomial logistic regression	None	Age; ethnicity; language; income; health condition/history; social support/social capital	Never used as opposed to use (Internet and computer)
Choi & DiNitto, 2013a	Internet and computer - communication, search for information, health and non-health related task	USA	n = 6680	Researcher administered survey (Secondary data)/Binary logistic regression analysis	None	Age; gender; ethnicity; education; income; health condition/history; social support/social capital	Use (Internet and computer)
Arcury et al., 2020	The Internet (via mobile technology or computer) - to improve access to health information and improve health self-management	USA	n = 200	Researcher administered survey/Chi square test/two-sample *t*-test	TAM	Age; gender; ethnicity; employment; education; income; health knowledge and attitudes; health condition/history; computer anxiety; social support/social capital; accessibility to technology	Use (Internet via mobile technology or computer)

Kim et al., 2021	Internet and computer - communication and social networking, reducing social isolation	USA	n = 77	Self- administered survey/Fisher's exact test & one-sided Sign test	None	Accessibility to technology	Use (Internet and computer)
Jung et al., 2010	The Internet and computer - increased autonomy, connectivity, self-efficacy, and search for information about health and hobbies	USA	n = 91	Researcher administered survey/Logistic regression analysis, Wald tests	Diffusion of innovation	Education; gender; income; experience; self-efficacy; computer anxiety; aging anxiety; belonging	Enrollment and use (computer and Internet)
Bertera et al., 2007	Telecare - allows home monitoring	USA	n = 85	Self-administered survey/Descriptive analysis	None	Perceived benefits (health)	Intention to use (Telecare)
Kim and Gray, 2016	Internet - to enhance social, psychological, and physical well-being	USA	n = 11	Semi-structured interviews (8–16 months after training program and actual use)/Coding and thematic analysis	None	Perceived benefits (Social participation; self- actualization; monetary); distrust; perceived cost; computer anxiety; self-efficacy; accessibility to technology	Use (Internet)
Berridge et al. , 2019	Sensor-based passive remote monitoring system - solution to the issue of insufficient numbers of elder care workers and family caregivers, to support aging in place and independent living	USA	n = 20 Note: total sample = 41, only themes from older adults were included)	Semi-structured interviews/Multi-step coding process of grounded theory - open coding and axial coding	None	Cultural background	Use (sensor-based remote monitoring system)
Berridge, 2017	Sensor-based passive remote monitoring system - solution to the issue of insufficient numbers of elder care workers and family caregivers, to support aging in place and independent living	USA	n = 20 Note: total sample = 41, only themes from older adults were included)	Semi-structured interviews/Multi-step coding process of grounded theory - open coding and axial coding	None	Perceived benefits (Social participation; self-actualization); perceived ease of use; perceived threat to autonomy; computer anxiety	Use sensor-based remote monitoring system)
Gallo et al., 2021	Electronic devices, e.g., cell phones, computer - facilitate in-person and virtual social participation to help reduce social isolation	USA	n = 48	Focus groups/Thematic analysis	None	Perceived benefits (Mobility; social participation); perceived risks; perceived cost; social support	Use (Electronic devices, e.g., cell phones, computer)
Nallam et al., 2020	Intelligent Voice Assistants (IVAs), e.g., Amazon Echo and Google Home - to support older adults at home for health-related and non-health-related tasks	USA	n = 10	Semi-structured interview/open coding and affinity diagramming	None	Perceived benefits (health); perceived risks; distrust; accessibility to technology	Intention to use (IVA)
Andonian, 2018	Computer - social participation with family and friends; leisure, financial management, and health care management and maintenance; banking access and bill payment; and health-related information	USA	n = 9	Survey and participatory action research (photovoice): focus groups, photo narratives/Thematic analysis Note: only qualitative data is relevant to RQ and included	None	Perceived benefits (self-actualization); freedom	Use (computer)
Latulipe et al., 2015	Patient portal - to access personal health records for economic and health care benefits	USA	n = 36 Note: Total sample = 52. Only themes from older adults were included	Interview/thematic analysis	None	Perceived benefits (health); perceived ease of use; perceived risks; perceived cost; interest; accessibility to technology	Use (Patient portal)
Seo et al., 2017	Internet and computer - communication, search for information, health and non-health related task	USA	n = 33	Focus groups/open coding	TAM	Perceived usefulness; perceived ease of use; perceived risks	Use (Internet and computer)
Choi et al., 2021	Tele-delivered behavioral activation (Tele-BA) via the Internet and laptop - improve both physical functioning and mood of patients	USA	n = 90	Randomized controlled trial, survey, interview/thematic analysis Note: only qualitative data is relevant to RQ and included	None	Perceived benefits (health); feeling productive and useful; hope; pride	Adoption (Tele-BA)

**Table B.1:** An overview of the personal factors related to technology use among low-income older adults

Dimensions of Determinant	Factor	Impact	Behavior and type of technology	Odds ratio (95% CI)/Percentage	P value	Study
Socio-demographic characteristics	*Age*					
	Age (older age group as opposed to younger age group) (group range not reported)	Positive	Never used as opposed to use (Internet and computer)	1.08 (1.06–1.11)	P < .001	Choi & DiNitto, 2013b*
	70-74 (as compared to 65–69 age group)	Negative	Use (Internet and computer)	0.62 (0.49–0.78)	P < .001	Choi & DiNitto, 2013a*
	75-79 (as compared to 65–69 age group)	Negative	Use (Internet and computer)	0.31 (0.25–0.38)	P < .001	Choi & DiNitto, 2013a*
	80-84 (as compared to 65–69 age group)	Negative	Use (Internet and computer)	0.22 (0.18–0.26)	P < .001	Choi & DiNitto, 2013a*
	≥85 (as compared to 65–69 age group)	Negative	Use (Internet and computer)	0.12 (0.09–0.16)	P < .001	Choi & DiNitto, 2013a*
	Age in years (55–64 years as compared to those aged 65 and older)	Positive	Use (Internet via mobile technology or computer)	65.50%	P < .01	Arcury et al., 2020*
	Age in years (60–64 years as compared to those aged 65 and older)	Positive	Use (Internet via mobile technology or computer)	61.90%	P < .01	Arcury et al., 2020*
	Age (group range not reported)	Non-significant	Enrollment and use (computer and Internet)	−0.01	Not reported	Jung et al., 2010 n.s.
	***Gender***					
	Male (as compared to female)	Positive	Enrollment and use (computer and Internet)	1.76 (β)	P < .05	Jung et al., 2010*
	Male (as compared to female)	Non-significant	Use (Internet via mobile technology or computer)	54.70%	P .67	Arcury et al., 2020 n.s.
	Female (as compared to male)	Non-significant	Use (Internet and computer)	0.97 (0.83–1.12)	–	Choi & DiNitto, 2013a n.s.
	Female (as compared to male)	Non-significant	Never used as opposed to use (Internet and computer)	0.94 (0.58–1.53)	–	Choi & DiNitto, 2013b n.s.
	***Ethnicity***					
	Black (as compared to White, Hispanic, Asian)	Negative	Use (Internet and mobile technology)	2.06 (1.12–3.80)	P = .02	Raven et al., 2018*
	Black (as opposed to non- Hispanic White)	Positive	Never used as opposed to use (Internet and computer)	5.13 (2.96–8.92)	P < .001	Choi & DiNitto, 2013b*
	Hispanic (as opposed to non- Hispanic white)	Positive	Never used as opposed to use (Internet and computer)	7.63 (3.28–17.74)	P < .001	Choi & DiNitto, 2013b*
	Non-Hispanic black (as compared to non-Hispanic white)	Negative	Use (Internet and computer)	0.41 (0.33–0.51)	P < .001	Choi & DiNitto, 2013a*
	Hispanic (as compared to non-Hispanic white)	Negative	Use (Internet and computer)	0.55 (0.39–0.78)	P < .001	Choi & DiNitto, 2013a*
	White (as compared to minority)	Positive	Use (Internet via mobile technology or computer)	67.50%	P < .01	Arcury et al., 2020*
	Minority versus white	Non-significant	Use (patient portal)	0.55 (0.19–1.60)	0.27	Arcury et al., 2017 n.s.
	***Language***					
	Spanish-speaking (as opposed to English speaking)	Non-significant	Never used as opposed to use (Internet and computer)	0.51 (0.06–4.49)	–	Choi & DiNitto, 2013b n.s.
	***Education***					
	Greater than high school versus high school or less	Positive	Use (patient portal); Use (Internet via mobile technology or computer)	5.75 (1.94–17.04); 75.7%	P < .01	Arcury et al., 2017*; Arcury et al., 2020*
	Less than high school/don't know/refused (as compared to those with at least a bachelor's degree)	Negative	Use (Internet and computer)	0.08 (0.06–0.10)	P < .001	Choi & DiNitto, 2013a*
	High school diploma or GED (as compared to those with at least a bachelor's degree)	Negative	Use (Internet and computer)	0.18 (0.14–0.24)	P < .001	Choi & DiNitto, 2013a*
	Some college or associate degree (as compared to those with at least a bachelor's degree)	Negative	Use (Internet and computer)	0.46 (0.37–0.57)	P < .001	Choi & DiNitto, 2013a*
	More years of education as compared to less years of education (range not reported)	Positive	Enrollment and use (computer and Internet)	0.13 (β)	P < .05	Jung et al., 2010*
	***Income***					
	Income-to-needs ratio (Having lower income as compared to higher income)	Positive	Never used as opposed to use (Internet and computer)	0.34 (0.24–0.48)	P < .001	Choi & DiNitto, 2013b*
	Having higher total income (in units of $10,000) (as compared to lower total income)	Positive	Use (Internet and computer)	1.01 (1.19–1.59)	(value not reported)	Choi & DiNitto, 2013a*
	Poverty level (higher income with greater than 200% of poverty level as compared to lower income with 200% of poverty or less)	Positive	Use (Internet via mobile technology or computer)	75%	P = .01	Arcury et al., 2020*
	Income (higher income as compared to lower income)	Non-significant	Enrollment and use (computer and Internet)	0.16	Not reported	Jung et al., 2010 n.s.
	***Employment***					
	Employed as compared to not employed	Non-significant	Use (Internet via mobile technology or computer)	62.50%	P .18	Arcury et al., 2020 n.s.
	***Health knowledge and attitudes***					
	General health literacy (adequate as compared to inadequate)	Positive	Use (Internet via mobile technology or computer)	83%	P < .01	Arcury et al., 2020*
	Medical decision-making attitude (do not prefer to rely on doctor's knowledge for medical decision making as compared to those who preferred to rely on their doctor's knowledge for medical decision making)	Positive	Use (Internet via mobile technology or computer)	68.20%	P < .01	Arcury et al., 2020*
	Number of health information sources (higher number versus lower number)	Positive	Use (Internet via mobile technology or computer)	Mean = 3.4 (SD = 1.5)	P < .01	Arcury et al., 2020*
	***Health condition/history***					
	Having activities of daily living (ADL) impairment (as compared to no ADL impairment)	Negative	Use (Internet and mobile technology)	Adjusted odds ratio 0.53 (0.31–0.92)	P = .02	Raven et al., 2018*
	Number of ADL/IADL impairment (higher number as compared to lower number)	Non-significant	Use (Internet and computer)	1.01 (0.97–1.04)	–	Choi & DiNitto, 2013a n.s.
		Non-significant	Never used as opposed to use (Internet and computer)	1.08 (0.98–1.17)	–	Choi & DiNitto, 2013b n.s.
	Cognitive impairment (Modified Mini-Mental (3MS) Examination)	Negative	Use (Internet and mobile technology)	0.56 (0.32–0.99)	P = .04	Raven et al., 2018*
	Executive function impairment	Negative	Use (Internet and mobile technology)	Adjusted odds ratio 0.49 (0.28–0.86)	P = .01	Raven et al., 2018*
	Moderate to high risk amphetamine use	Negative	Use (Internet and mobile technology)	Adjusted odds ratio 0.27 (0.10–0.72)	P = .01	Raven et al., 2018*
	Dementia/Alzheimer's disease (as compared to no dementia/Alzheimer's disease)	Negative	Use (Internet and computer)	0.37 (0.20–0.66)	P < .001	Choi & DiNitto, 2013a*
	Depression diagnosis (as opposed to no depression diagnosis)**	Negative	Never used as opposed to use (Internet and computer)	0.62 (0.39–0.98)	P < .05	Choi & DiNitto, 2013b*
		Negative	Use (Internet and computer)	0.83 (0.70–0.98)	P < .05	Choi & DiNitto, 2013a*
	Anxiety diagnosis (as opposed to no anxiety diagnosis)	Negative	Use (Internet and computer)	0.79 (0.65–0.97)	P < .05	Choi & DiNitto, 2013a*
		Non-significant	Never used as opposed to use (Internet and computer)	1.11 (0.63–1.96)		Choi & DiNitto, 2013b n.s.
	Good to excellent health	Non-significant	Use (Internet and mobile technology)	1.63 (0.96–2.78)	P = .07	Raven et al., 2018 n.s.
	Heavy drinking	Non-significant	Use (Internet and mobile technology)	0.58 (0.32–1.02)	P = .06	Raven et al., 2018 n.s.
	Charlson Comorbidity Index (worse health of 5 or more chronic conditions versus fewer than 5)	Non-significant	Use (patient portal); Use (Internet via mobile technology or computer)	2.43 (0.65–9.13) Mean = 6.1 (SD = 2.6)	P = .18 P = .37	Arcury et al., 2017 n.s. Arcury et al., 2020 n.s.
	Number of medical conditions (higher number as compared to lower number)	Non-significant	Never used as opposed to use (Internet and computer)	0.92 (0.79–1.07)	–	Choi & DiNitto, 2013b n.s.
	Number of chronic illnesses (higher number as compared to lower number)	Non-significant	Use (Internet and computer)	1.01 (0.97–1.05)	–	Choi & DiNitto, 2013a n.s.
	SF-12 Mental Component Score subscale (higher score compared to lower score)	Non-significant	Use (Internet via mobile technology or computer)	Mean = 49.7 (SD = 11.4)	P .55	Arcury et al., 2020 n.s.
	Physical Component Score subscale (higher score compared to lower score)	Non-significant	Use (Internet via mobile technology or computer)	Mean = 37.8 (SD = 12.5)	P .84	Arcury et al., 2020 n.s.
	***Cultural Background +***					
	Values Made Visible	–	Use (sensor-based remote monitoring system)	–	–	Berridge et al., 2019 (q)
	The Role of the Cultural Navigator	–	Use (sensor-based remote monitoring system)	–	–	Berridge et al., 2019 (q)
	***Experience***					
	Computer experience (greater computer experience as compared to lesser experience)	Positive	Enrollment and use (computer and Internet)	1.09	P < .06 (Marginally significant)	Jung et al., 2010*
	Internet use frequency (at least once a day versus less than once a day)	Positive	Use (patient portal)	7.08 (2.55–19.67)	P < .01	Arcury et al., 2017*
**Cognitive Evaluations**	***Self-efficacy***					
	Computer self-efficacy/Self- efficacy (higher versus lower)	Positive	Enrollment and use (computer and Internet)	1.12 (β)	P < .05	Jung et al., 2010*
		Non-significant	Use (Internet and computer)	1.05 (0.99–1.11)	Not reported	Choi & DiNitto, 2013a n.s.
	Low literacy (due to years of education)	–	Use (Internet)	–	–	Kim and Gray, 2016 (q)
	(Dis)ability (in terms of physical ability and know-how)	–	Use (Electronic device)	–	–	Gallo et al., 2021 (q)
	Limited proficiency	–	Use (Internet)	–	–	Kim and Gray, 2016 (q)
	***Perceived Benefits (health)***					
	Allows me to communicate with clinician from home	–	Intention to use (Telecare)	97%	–	Bertera et al., 2007^
	Calls for medical help when needed	–	Intention to use (Telecare)	96%	–	Bertera et al., 2007^
	Helps me manage medications	–	Intention to use (Telecare)	90%	–	Bertera et al., 2007^
	Is something I wear daily to signal a nurse I'm OK	–	Intention to use (Telecare)	89%	–	Bertera et al., 2007^
	Monitor if/when I fall in my home	–	Intention to use (Telecare)	88%	–	Bertera et al., 2007^
	Detects if I'm able to get around as usual in my home	–	Intention to use (Telecare)	86%	–	Bertera et al., 2007^
	Sends health information (e.g. blood glucose, blood pressure readings) to my doctor	–	Intention to use (Telecare)	85%	–	Bertera et al., 2007^
	Allows me to get health information from the Internet	–	Intention to use (Telecare)	82%	–	Bertera et al., 2007^
	Allows a nurse to check on me with a camera when I am unwell	–	Intention to use (Telecare)	68%	–	Bertera et al., 2007^
	Support them with other health tasks	–	Intention to use (IVA)	–	–	Nallam et al., 2020 (q)
	Obtaining emergency assistance	–	Intention to use (IVA)	–	–	Nallam et al., 2020 (q)
	Digital Archiving and Analysis	–	Use (Patient portal)	–	–	Latulipe et al., 2015 (q)
	Neutral Communication Medium	–	Use (Patient portal)	–	–	Latulipe et al., 2015 (q)
	Benefits of psychoeducation	–	Adoption (Tele-BA)	–	–	Choi et al., 2021 (q)
	Importance of and gratitude for working with a counselor	–	Adoption (Tele-BA)	–	–	Choi et al., 2021 (q)
	Benefits of activity planning	–	Adoption (Tele-BA)	–	–	Choi et al., 2021 (q)
	Understanding of behavior and mood connection	–	Adoption (Tele-BA)	–	–	Choi et al., 2021 (q)
	Positive effects of increased activity on health and self- enjoyment	–	Adoption (Tele-BA)	–	–	Choi et al., 2021 (q)
	Complete medical records	–	Use (patient portal)	–	–	Latulipe et al., 2015 (q)
	Contextualized medical advice	–	Use (patient portal)	–	–	Latulipe et al., 2015 (q)
	***Perceived Benefits (Mobility)***					
	Using technology for mobility	–	Use (electronic device)	–	–	Gallo et al., 2021 (q)
	***Perceived Benefits (Social Participation)***					
	Enhanced social connections	–	Use (Internet)	–	–	Kim and Gray, 2016 (q)
	Technology-mediated social participation.	–	Use (electronic device)	–	–	Gallo et al., 2021 (q)
	Strategic ‘misuse’ to combat social isolation	–	Use (Sensor-based remote monitoring system)	–	–	Berridge, 2017 (q)
	***Perceived Benefits (Self-Actualization)***			–	–	
	Development of life skills	–	Use (Internet)	–	–	Kim and Gray, 2016 (q)
	Recognition of personal growth and change	–	Use (Computer)	–	–	Andonian, 2018 (q)
	Contribution and engagement in relationships and the global community	–	Use (Computer)	–	–	Andonian, 2018 (q)
	Adopting is "the right thing to do"	–	Use (Sensor-based remote monitoring system)	–	–	Berridge, 2017 (q)
	***Perceived Benefits (Monetary)***					
	Monetary benefits	–	Use (Internet)	–	–	Kim and Gray, 2016 (q)
	***Perceived Usefulness***	–	Use (Internet and computer)	–	–	Seo et al., 2017 (q)
	***Perceived Ease of Use***			–	–	
	Low level of perceived ease of use	–	Use (Internet and computer)	–	–	Seo et al., 2017 (q)
	Usability issues	–	Use (patient portal)	–	–	Latulipe et al., 2015 (q)
	Reliance of the system on one's ability to communicate with the operator	–	Adoption (Sensor-based remote monitoring system)	–	–	Berridge, 2017 (q)
	***Perceived risks***					
	Privacy concerns	–	Use (Internet and computer)	–	–	Seo et al., 2017 (q)
	Confidentiality risks - concerns about privacy	–	Intention to use (IVA)	–	–	Nallam et al., 2020 (q)
	Security concerns	–	Use (Internet and computer)	–	–	Seo et al., 2017 (q)
	Privacy and security	–	Use (patient portal)	–	–	Latulipe et al., 2015 (q)
	Perceived lack of safety	–	Use (Electronic device)	–	–	Gallo et al., 2021 (q)
	***Distrust +***					
	Suspicion or distrust of governmental or social programs	–	Use (Internet)	–	–	Kim and Gray, 2016 (q)
	Concerns about receiving trusted information - trusting the information provided by an IVA	–	Intention to use (IVA)	–	–	Nallam et al., 2020 (q)
	***Perceived threat to autonomy +***					
	Disruptive assumptions about ‘normal’ habitual behavior	–	Discontinued use (sensor-based remote monitoring system)	–	–	Berridge, 2017 (q)
	‘Modifying behavior to avoid alert signals’	–	Use (sensor-based remote monitoring system)	–	–	Berridge, 2017 (q)
	‘Misuse’ to exercise control	–	Use (sensor-based remote monitoring system)	–	–	Berridge, 2017 (q)
	***Perceived cost +***					
	Cost	–	Use (Electronic device)	–	–	Gallo et al., 2021 (q)
	Costs of broadband subscription	–	Use (Internet)	–	–	Kim and Gray, 2016 (q)
	Provider Face Time (fear that use of the patient portal could eventually replace face-to-face visits)	–	Use (Patient portal)	–	–	Latulipe et al., 2015 (q)
**Affective Responses**	***Computer anxiety***					
	Computer stress	Negative	Use (Internet via mobile technology or computer)	41.10%	P < .01	Arcury et al., 2020*
	Computer anxiety	Negative	Enrollment and use (computer and Internet)	−1.28 (β)	P < .01	Jung et al., 2010*
	Fear of technology	–	Use (Internet)	–	–	Kim and Gray, 2016 (q)
	Fear or misconceptions about cyber security	–	Use (Internet)	–	–	Kim and Gray, 2016 (q)
	Uncomfortable, fearful and ‘spooked’ (by QuietCare’)	–	Use (sensor-based remote monitoring system)	–	–	Berridge, 2017 (q)
	***Aging anxiety***	Negative	Enrollment and use (computer and Internet)	−1.35 (β)	P < .01	Jung et al., 2010*
	***Interest***	–	Use (Patient portal)	–	–	Latulipe et al., 2015 (q)
	***Freedom****to express interests, values, and identity*	–	Use (Computer)	–	–	Andonian, 2018 (q)
	***Feeling productive and useful***	–	Adoption (Tele-BA)	–	–	Choi et al., 2021 (q)
	***Hope****for the future*	–	Adoption (Tele-BA)	–	–	Choi et al., 2021 (q)
	***Pride****in doing tele-sessions*	–	Adoption (Tele-BA)	–	–	Choi et al., 2021 (q)
	***Belonging***					
	Total belonging (neighbourhood belonging)	Non-significant	Enrollment and use (computer and Internet)	0.05	Not reported	Jung et al., 2010 n.s.
	Feelings of belonging to senior center	Non-significant	Enrollment and use (computer and Internet)	−0.38	Not reported	Jung et al., 2010 n.s.
	Perceived (feelings of) social support	Non-significant	Enrollment and use (computer and Internet)	−0.40	Not reported	Jung et al., 2010 n.s.

Note. *significant, n.s. nonsignificant, ** contradictory, ^no correlation conducted, (β) logit coefficients, + aggregated and self-coded, (q) qualitative data.

**Table:** 

Dimensions of Determinant	Factor	Impact	Behavior and Type of Technology	Odds ratio (95% CI)/Percentage	P value	Study
Social Environment	*Social Support/Social Capital*					
	Not currently married versus married	Negative	Use (Patient portal)	0.17 (0.06–0.52)	P = .001	Arcury et al., 2017*
	Married (as compared to not married)	Positive	Use (Internet via mobile technology or computer)	64.30%	P < .01	Arcury et al., 2020*
	Living with a spouse (as compared to not living with a spouse) **	Positive	Use (Internet and computer)	1.38 (1.19–1.59)	P < .001	Choi & DiNitto, 2013a*
	Living alone (as opposed to living with someone) **	Negative	Never used as opposed to use (Internet and computer)	0.78 (0.48–1.24)	P < .05	Choi & DiNitto, 2013b*
	Have at least 1 living child (as compared to not having at least 1 living child)	Positive	Use (Internet and computer)	1.47 (1.18–1.84)	P < .001	Choi & DiNitto, 2013a*
	Have at least 1 living sibling (as compared to not having at least 1 living sibling)	Negative	Use (Internet and computer)	0.81(0.68–0.99)	P < .05	Choi & DiNitto, 2013a*
	Support from family and friends	–	Use (Electronic device)	–	–	Gallo et al., 2021 (q)
	Household structure (1 person as compared to 2/3 or more persons)	–	Internet use (via mobile technology or computer)	45.70%	P .08	Arcury et al., 2020 n.s.
	Care partner (having a care partner as compared to not having a care partner)	–	Internet use (via mobile technology or computer)	48.40%	P .09	Arcury et al., 2020 n.s.
	Worked for pay last month (as compared to have not worked for pay last month)	Positive	Use (Internet and computer)	1.65 (1.34–2.03)	P < .001	Choi & DiNitto, 2013a*
	Did formal volunteering last month (as compared to did not involve in formal volunteering last month)	Positive	Use (Internet and computer)	1.33 (1.09–1.62)	P < .01	Choi & DiNitto, 2013a*
	Visited family/friend last month (as compared to have not visited family/friend last month)	Positive	Use (Internet and computer)	1.25 (1.03–1.52)	P < .05	Choi & DiNitto, 2013a*
	Attended religious service last month (as compared to did not attended religious service last month)	Negative	Use (Internet and computer)	0.82 (0.73–0.94)	P < .05	Choi & DiNitto, 2013a*
	Attended clubs/classes/organized activities last month (as compared to have not attended clubs/classes/organized activities last month)	Positive	Use (Internet and computer)	1.73 (1.46–2.05)	P < .001	Choi & DiNitto, 2013a*
	Went out for enjoyment last month (as compared to have not gone out for enjoyment last month)	Positive	Use (Internet and computer)	1.69 (1.36–2.08)	P < .001	Choi & DiNitto, 2013a*
	Provided informal caregiving last month (as compared to have not provided informal caregiving last month)	–	Use (Internet and computer)	1.00 (0.83–1.19)	Not reported	Choi & DiNitto, 2013a n.s.
**Technological Environment**	***Accessibility of technology +***					
	Number of e-devices (Having two or more e-devices in their homes as compared to those with one or no home e-devices	Positive	Use (Internet via mobile technology or computer)	82.10%	P < .01	Arcury et al., 2020*
	Lack of technology	–	Use (patient portal)	–	–	Latulipe et al., 2015 (q)
	Individual device needs	–	Use (Internet)	–	–	Kim and Gray, 2016 (q)
	Access barriers - maintaining the technical resources needed to support an IVA	–	Intention to use (IVA)	–	–	Nallam et al., 2020 (q)
	Problems with broadband services.	–	Use (Internet)	–	–	Kim and Gray, 2016 (q)
	Preference for wi-fi	–	Use (Internet)	–	–	Kim and Gray, 2016 (q)
	Technical support needs	–	Use (Internet)	–	–	Kim and Gray, 2016 (q)
	Computer and Internet use training program	Positive	Use (Email/Internet)	Pretest = 5.2%, Posttest = 40.3%	P < .0005	Kim et al., 2021*
**Physical Environment**	***Housed****(as compared to those who have not regained housing/homeless)*	Positive	Use (Internet and mobile technology)	AOR (95% CI) 3.81 (1.77–8.21)	P < .001	Raven et al., 2018*
	***Urban****(as compared to rural clinic)*	Positive	Use (patient portal)	12.21 (3.05–48.87)	P < .01	Arcury et al., 2017*
**Organizational Characteristics**	***Insurance status***					
	Health insurance - private insurance versus none	–	Use (patient portal)	0.73 (0.11–5.07)	0.86	Arcury et al., 2017 n.s.
	Health insurance- Government insurance versus none	–	Use (patient portal)	0.61 (0.09–4.10)	Not reported	Arcury et al., 2017 n.s.

Note. *significant, n.s. nonsignificant, ** contradictory, ^no correlation conducted, (β) logit coefficients, + aggregated and self-coded, (q) qualitative data.

**Table C.1:** An overview of technology type, technology ownership and usage patterns

Type of technology	Technology	Technology Ownership	Behavior	Patterns of use	Reference
ICT	The Internet and mobile technology	Yes (73.6%)	Use	Mobile phones: Contact relatives and friends, medical personnel, social service agencies, shelters or other housing providers, potential employers, and potential landlords The Internet: Read or send email; get news online; watch a video, download a music file, or play a game; browse the internet for fun; look for information (about a shelter or place to live, hobby or interest, health or medical information, job); check social networking sites	Raven et al., 2018
	The Internet and computer	Yes - cell phone (80.19%) - computer (68.5%)	Use	To send emails or text messages; to pay bills or do banking; to obtain information about health conditions for self or others; to shop for groceries or personal items; to order or refill prescriptions; to contact any medical provider; to handle Medicare or other health insurance matters	Choi & DiNitto, 2013 (a)
	The Internet and computer	Yes (95.2%)	Never used as opposed to use	Send/receive email; research health-related information and information about other topics; do banking/paying bills online; play games; read papers/news, magazines, and books; buy product online; watch videos; use social network or dating site	Choi & DiNitto (b), 2013
	The Internet and computer	No (access was through CyberCafe′ at the senior centre)	Enrolment and use	(Not reported)	Jung et al., 2010
	The Internet (via mobile technology or computer)	Yes (72%)	Use	(Not reported)	Arcury et al., 2020
	The Internet and computer	Yes - computer (97.4%) - Internet (70.1%)	Use	To access email	Kim et al., 2021
	The Internet	No (loaned laptop and access to free broadband services for 12 months)	Use	(Not reported)	Kim and Gray, 2016
	Computer and Internet (including tablets, laptops, and smartphones)	Yes (88.9%)	Use	Engage in activities that express their deeply held interests and values (such as Jewish identity, writing, virtual travel, and learning); free access to information to complete paperwork online; to support learning in varied occupations; to connect with friends and relatives; to enlarge their sphere of knowledge	Andonian, 2018
	Electronic devices such as: Cell phone, smartphone, computing devices (computer at home/tablet/e-reader/computer with internet at cybercafe etc) (ICT)	Yes - cell phone/smartphone (87%) - computer/tablet (43.7%)	Use	To check the news and weather; search for information; learn English; call, message, and video chat family and friends; watch Netflix and YouTube; play games; call rideshare services; check directions and bus schedules; call the Metropolitan Transportation Authority (Metro) for bus routes and schedules; keep entertained while traveling	Gallo et al., 2021
	The Internet and computer or other digital technologies such as tablet or cell phone	(Not reported)	Adoption and use	To search for information; online banking and bill pay, keep in touch with family members, access medical records, and play games	Seo et al., 2017
Digital health technology	Patient portal	(Not reported)	Use	View lab or test results; send message to doctor or nurse; make or change an appointment; refill prescriptions; find information about a health issue	Arcury et al., 2017
	Patient portal	(Not reported)	Adoption and use (Pre/post use)	Look at lab results, schedule appointments, send questions to the healthcare provider and renew prescriptions	Latulipe et al., 2015
	Telecare	(Not reported)	Acceptance (Pre-use)	Potentially to communicate with clinician from home; call for medical help when needed; manage medications; signal a nurse I'm OK; monitor if/when I fall in my home; detects if I'm able to get around as usual in my home; sends health information (e.g., blood glucose, blood pressure readings) to my doctor; to get health information from the Internet	Bertera et al., 2007
	Tele-delivered behavioral activation (Tele-BA)	No (loaned laptop and internet connection)	Acceptance and use	Attend tele-sessions, learn and practice behavioral activation (To improve self-care management of physical and mental health; engage in self-enrichment/self-enjoyment, improve living environment and daily routine; and to increase social connectedness)	Choi et al., 2021
Smart sensor	Sensor-based passive remote monitoring system	Yes	Adoption and use	Passive use	Berridge et al., 2019
	Sensor-based passive remote monitoring system	Yes	Adoption and use	‘Misuse’ to combat social isolation (manipulate the technology to receive calls from the operators); ‘Misuse’ to exercise control (manoeuvring to outsmart the system to avoid wasting time with unnecessary hospital care and troubling their emergency contacts)	Berridge, 2017
AI	Intelligent Voice Assistants (IVAs)	No (interaction was through prototype)	Acceptance (Pre-use)	Assist with general decisions (e.g., recommending a medication) based on a verified fact; research on illnesses or medications; manage health; obtain emergency assistance	Nallam et al., 2020

## References

[1] World Health Organization (2020).

[2] Ma Q., Chan A.H.S., Teh P.-L. (2021). Insights into older adults' technology acceptance through meta-analysis. Int. J. Hum. Comput. Interact..

[3] Wagner N., Hassanein K., Head M. (2010). Computer use by older adults: a multi-disciplinary review. Comput. Hum. Behav..

[4] Peek S.T.M., Wouters E.J.M., van Hoof J., Luijkx K.G., Boeije H.R., Vrijhoef H.J.M. (2014). Factors influencing acceptance of technology for aging in place: a systematic review. Int. J. Med. Inf..

[5] Wang J., Fu Y., Lou V., Tan S.Y., Chui E. (2021). A systematic review of factors influencing attitudes towards and intention to use the long-distance caregiving technologies for older adults. Int. J. Med. Inf..

[6] Luoma-Halkola H., Häikiö L. (2020). Independent living with mobility restrictions: older people's perceptions of their out-of-home mobility. Ageing Soc..

[7] Ibarra F., Baez M., Cernuzzi L., Casati F. (2020). A systematic review on technology-supported interventions to improve old-age social wellbeing: loneliness, social isolation, and connectedness. J. Healthc. Eng..

[8] Sen K., Prybutok G., Prybutok V. (2022). The use of digital technology for social wellbeing reduces social isolation in older adults: a systematic review. SSM - Popul. Health.

[9] Aggarwal B., Xiong Q., Schroeder-Butterfill E. (2020).

[10] Baishya K., Samalia H.V. (2020). Extending unified theory of acceptance and use of technology with perceived monetary value for smartphone adoption at the bottom of the pyramid. Int. J. Inf. Manag..

[11] Evans M.C., Bazargan M., Cobb S., Assari S. (2020). Mental and physical health correlates of financial difficulties among african-American older adults in low-income areas of los angeles. Front. Publ. Health.

[12] Cohen-Mansfield J., Hazan H., Lerman Y., Shalom V. (2016). Correlates and predictors of loneliness in older-adults: a review of quantitative results informed by qualitative insights. Int. Psychogeriatr..

[13] Gonyea J.G., Curley A., Melekis K., Levine N., Lee Y. (2018). Loneliness and depression among older adults in urban subsidized housing. J. Aging Health.

[14] Chen Q., Amano T., Park S., Kim B. (2019). Home and community-based services and life satisfaction among homebound and poor older adults. J. Gerontol. Soc. Work.

[15] Dudley N., Rauch L., Adelman T., Canham D. (2021). Addressing needs of older adults in low-income independent living facilities in community health. Innov. Aging.

[16] Chen R., Schulz P.J. (2016). The effect of information communication technology interventions on reducing social isolation in the elderly: a systematic review. J. Med. Internet Res..

[17] McInerney M., McCormack G., Mellor J.M., Sabik L.M. (2022). Association of medicaid expansion with medicaid enrollment and health care use among older adults with low income and chronic condition limitations. JAMA Health Forum.

[18] Showell C., Cummings E., Turner P. (2017). The invisibility of disadvantage: why do we not notice?. Stud. Health Technol. Inf..

[19] Bertera E.M., Tran B.Q., Wuertz E.M., Bonner A. (2007). A study of the receptivity to telecare technology in a community-based elderly minority population. J. Telemed. Telecare.

[20] Hargittai E., Piper A.M., Morris M.R. (2019). From internet access to internet skills: digital inequality among older adults. Univers. Access Inf. Soc..

[21] Kavandi H., Jaana M. (2020). Factors that affect health information technology adoption by seniors: a systematic review. Health Soc. Care Community.

[22] Wilson S.A., Byrne P., Rodgers S.E., Maden M. (2022). A systematic review of smartphone and tablet use by older adults with and without cognitive impairment. Innov. Aging.

[23] Yap Y.-Y., Tan S.-H., Choon S.-W. (2022). Elderly's intention to use technologies: a systematic literature review. Heliyon.

[24] Yoon H., Jang Y., Vaughan P.W., Garcia M. (2020). Older adults' internet use for health information: digital divide by race/ethnicity and socioeconomic status. J. Appl. Gerontol..

[25] Bandura A. (1986).

[26] Bandura A. (1989). Regulation of cognitive processes through perceived self-efficacy. Dev. Psychol..

[27] Maslow A.H. (1943). A theory of human motivation. Psychol. Rev..

[28] Maslow A.H. (1954).

[29] Chetty R., Stepner M., Abraham S., Lin S., Scuderi B., Turner N., Bergeron A., Cutler D. (2016). The association between income and life expectancy in the United States, 2001–2014: association between income and life expectancy in the United States. JAMA, J. Am. Med. Assoc..

[30] University of Michigan Health and Retirement Study (2017).

[31] The Institute for Fiscal Studies (2020).

[32] Hawley-Hague Helen, Boulton Elisabeth, Hall Alex, Pfeiffer Klaus, Todd Chris (2014). Older adults' perceptions of technologies aimed at falls prevention, detection or monitoring: a systematic review. Int. J. Med. Inf..

[33] Hirvonen N., Enwald H., Känsäkoski H., Eriksson-Backa K., Nguyen H., Huhta A.-M., Huvila I. (2020). Older adults' views on eHealth services: a systematic review of scientific journal articles. Int. J. Med. Inf..

[34] Hill Courtney M., Tseng Ashley S., Holzhauer, Katherine, Littman, Alyson J., Jones-Smith, Jessica C. (2023). Association between health care access and food insecurity among lower-income older adults with multiple chronic conditions in Washington State, USA. Publ. Health Nutr..

[35] Crist Katie, Full, Kelsie M., Linke Sarah, Tuz-Zahra Fatima, Bolling Khalisa, Lewars Brittany, Liu Chenyu, Shi Yuyan, Rosenberg Dori, Jankowska Marta, Benmarhnia Tarik, Natarajan Loki (2022). Health effects and cost-effectiveness of a multilevel physical activity intervention in low-income older adults; results from the PEP4PA cluster randomized controlled trial. Int. J. Behav. Nutr. Phys. Activ..

[36] Choi Namkee G., Marti C., Bruce Nathan, Martha L., Hegel Mark T., Wilson Nancy L., Kunik Mark E. (2014). Six-month postintervention depression and disability outcomes of in-home telehealth problem-solving therapy for depressed, low-income homebound older adults. Depress. Anxiety.

[37] Shi Jian-gang, Liu Menglan, Fu Guoqiang, Dai Xingying (2023). Internet use among older adults: determinants of usage and impacts on individuals' well-being. Comput. Hum. Behav..

[38] Jones Deborah J., Anton Margaret, Zachary Chloe, Pittman Sarah, Turner Patrick, Forehand Rex, Khavjou Olga (2016). A review of the key considerations in mental health services research: a focus on low-income children and families. Couple and Fam. Psychol..

[39] The World Bank (2022). https://www.worldbank.org/en/news/factsheet/2022/05/02/fact-sheet-an-adjustment-to-global-poverty-lines.

[40] Organisation for Economic Co-operation and Development (2016).

[41] Davis F.D. (1989). Perceived usefulness, perceived ease of use, and user acceptance of information technology. MIS Q..

[42] Venkatesh V., Morris M.G., Davis G.B., Davis F.D. (2003). User acceptance of information technology: toward a unified view. MIS Q..

[43] Chen K., Chan A.H.S. (2014). Gerontechnology acceptance by elderly Hong Kong Chinese: a senior technology acceptance model (STAM). Ergonomics.

[44] Venkatesh V., Thong J.Y.L., Xu X. (2012). Consumer acceptance and use of information technology: extending the unified theory of acceptance and use of technology. MIS Q..

[45] Bandura A. (2009). Media Effects.

[46] Bandura A. (1991). Social cognitive theory of self-regulation. Organ. Behav. Hum. Decis. Process..

[47] Bandura A. (1977). Self-efficacy: toward a unifying theory of behavioral change. Psychol. Rev..

[48] Bennett G.G., Steinberg D., Askew S., Levine E., Foley P., Batch B.C., Svetkey L.P., Bosworth H.B., Puleo E.M., Brewer A., DeVries A., Miranda H. (2018). Effectiveness of an app and provider counseling for obesity treatment in primary care. Am. J. Prev. Med..

[49] Yang H.-L., Lin S.-L. (2019). The reasons why elderly mobile users adopt ubiquitous mobile social service. Comput. Hum. Behav..

[50] Page M.J., McKenzie J.E., Bossuyt P.M., Boutron I., Hoffmann T.C., Mulrow C.D., Shamseer L., Tetzlaff J.M., Akl E.A., Brennan S.E., Chou R., Glanville J., Grimshaw J.M., Hróbjartsson A., Lalu M.M., Li T., Loder E.W., Mayo-Wilson E., McDonald S., Moher D. (2021).

[51] Li J., Brar A. (2022). The use and impact of digital technologies for and on the mental health and wellbeing of Indigenous people: a systematic review of empirical studies. Comput. Hum. Behav..

[52] CASP (2012).

[53] HEBW (2013). https://studylib.net/doc/7216606/case-control-study-checklist--2-.

[54] Schlackl F., Link N., Hoehle H. (2022). Antecedents and consequences of data breaches: a systematic review. Inf. Manag..

[55] Arcury T.A., Sandberg J.C., Melius K.P., Quandt S.A., Leng X., Latulipe C., Miller D.P., Smith D.A., Bertoni A.G. (2020). Older adult internet use and eHealth literacy. J. Appl. Gerontol..

[56] Choi N.G., DiNitto D.M. (2013). The digital divide among low-income homebound older adults: internet use patterns, eHealth literacy, and attitudes toward computer/Internet use. J. Med. Internet Res..

[57] Arcury T.A., Quandt S.A., Sandberg J.C., Miller David P J., Latulipe C., Leng X., Talton J.W., Melius K.P., Smith A., Bertoni A.G. (2017). Patient portal utilization among ethnically diverse low income older adults: observational study. JMIR Med. Info..

[58] Berridge C. (2017). Active subjects of passive monitoring: responses to a passive monitoring system in low-income independent living. Ageing Soc..

[59] Berridge C., Chan K.T., Choi Y. (2019). Sensor-based passive remote monitoring and discordant values: qualitative study of the experiences of low-income immigrant elders in the United States. JMIR MHealth and UHealth.

[60] Choi N.G., DiNitto D.M. (2013). Internet use among older adults: association with health needs, psychological capital, and social capital. J. Med. Internet Res..

[61] Choi N.G., Caamano J., Vences K., Marti C.N., Kunik M.E. (2021). Acceptability and effects of tele-delivered behavioral activation for depression in low-income homebound older adults: in their own words. Aging Ment. Health.

[62] Jung Y., Peng W., Moran M., Jin S.-A.A., McLaughlin M., Cody M., Jordan-Marsh M., Albright J., Silverstein M. (2010). Low-income minority seniors' enrollment in a cybercafé: psychological barriers to crossing the digital divide. Educ. Gerontol..

[63] Kim J., Gray J.A., Ciesla J.R., Yao P. (2021). The impact of an internet use promotion programme on communication, internet use, and the extent of social networks among low-income older adults. Ageing Int..

[64] Nallam P., Bhandari S., Sanders J., Martin-Hammond A. (2020). A question of access: exploring the perceived benefits and barriers of intelligent Voice assistants for improving access to consumer health resources among low-income older adults. Gerontol. Geriatric Med..

[65] Seo H., Erba J., Geana M., Lumpkins C. (2017). Calling doctor Google? Technology adoption and health information seeking among low-income african-American older adults. J. Publ. Interest Commun..

[66] Raven M.C., Kaplan L.M., Rosenberg M., Tieu L., Guzman D., Kushel M. (2018). Mobile phone, computer, and internet use among older homeless adults: results from the HOPE HOME cohort study. JMIR MHealth and UHealth.

[67] Andonian L.C. (2018). Meanings and experiences associated with computer use of older immigrant adults of lower socioeconomic status: les sens et les expériences associés à l’utilisation des ordinateurs chez les immigrants âgés ayant un faible statut socioéconomique. Can. J. Occup. Ther..

[68] Gallo H.B., Marshall L.W., Levy-Storms L., Wilber K.H., Loukaitou-Sideris A. (2021). Voices of experience: what do low-income older adults tell us about mobility, technology, and social participation?. J. Appl. Gerontol..

[69] Kim J., Gray J. (2016). Qualitative evaluation of an intervention program for sustained internet use among low-income older adults. Ageing Int..

[70] Latulipe C., Gatto A., Nguyen H., Miller D., Quandt S., Bertoni A., Smith A., Arcury T. (2015). Design considerations for patient portal adoption by low-income, older adults. Conf. Human Factors in Comput. Syst..

[71] Schaie K. Warner, Schooler Carmi (1988). Social Structure and Aging.

[72] Rogers E.M. (2003). https://ebookcentral.proquest.com/lib/monash/detail.action?docID=4935198.

[73] Rojas M., Méndez A., Watkins-Fassler K. (2023). The hierarchy of needs empirical examination of Maslow's theory and lessons for development. World Dev..

[74] Kenrick D.T., Griskevicius V., Neuberg S.L., Schaller M. (2010). Renovating the pyramid of needs: contemporary extensions built upon ancient foundations. Perspect. Psychol. Sci..

[75] McLeod S. (2007). Maslow's hierarchy of needs. Simply Psychol.

[76] Bandura A. (2012). On the functional properties of perceived self-efficacy revisited. J. Manag..

[77] Ma Q., Chen K., Chan A.H.S., Teh P.-L. (2015).

[78] Sakaguchi-Tang D.K., Bosold A.L., Choi Y.K., Turner A.M. (2017). Patient portal use and experience among older adults: systematic review. JMIR Med. Info..

[79] Musselwhite C., Haddad H. (2010). Mobility, accessibility and quality of later life. Q. Ageing.

[80] Wan X., Lighthall N.R., Xie R. (2022). Consistent and robust predictors of Internet Use among older adults over time identified by machine learning. Comput. Hum. Behav..

[81] Ancker J.S., Barrón Y., Rockoff M.L., Hauser D., Pichardo M., Szerencsy A., Calman N. (2011). Use of an electronic patient portal among disadvantaged populations. J. Gen. Intern. Med. : JGIM.

[82] Chen K. (2020). Why do older people love and hate assistive technology? ‒ an emotional experience perspective. Ergonomics.

[83] Jung B., Kim H., Lee S.H., Shawn) (2022). The impact of belongingness and graphic-based emoticon usage motives on emoticon purchase intentions for MIM: an analysis of Korean KakaoTalk users. Online Inf. Rev..

[84] Thielke S., Harniss M., Thompson H., Patel S., Demiris G., Johnson K. (2012). Maslow's hierarchy of human needs and the adoption of health-related technologies for older adults. Ageing Int..

[85] Darcy J., Hoffman C.-A. (2003).

[86] Pang W.Y.J., Cheng L. (2023). Acceptance of gamified virtual reality environments by older adults. Educ. Gerontol..

[87] Xu W., Liang H.-N., Yu K., Wen S., Baghaei N., Tu H. (2023). Acceptance of virtual reality exergames among Chinese older adults. Int. J. Hum. Comput. Interact..

[88] Stein M.-K., Newell S., Wagner E.L., Galliers R.D. (2015). Coping with information technology: mixed emotions, vacillation, and nonconforming use patterns. MIS Q..

[89] Pauketat J.V.T., Anthis J.R. (2022). Predicting the moral consideration of artificial intelligences. Comput. Hum. Behav..

[90] Beaudry A., Pinsonneault A. (2010). The other side of acceptance: studying the direct and indirect effects of emotions on information technology use. MIS Q..

[91] Zhang P. (2013). The affective response model: a theoretical framework of affective concepts and their relationships in the ICT context. MIS Q..

[92] Carstensen L.L., Isaacowitz D.M., Charles S.T. (1999). Taking time seriously: a theory of socioemotional selectivity. Am. Psychol..

[93] Watkins I., Xie B. (2014). eHealth literacy interventions for older adults: a systematic review of the literature. J. Med. Internet Res..

[94] Alvesson M., Sandberg J. (2014). Habitat and habitus: boxed-in versus box-breaking research. Organ. Stud..

[95] Singh N., Misra R., Singh S., Rana N.P., Khorana S. (2022). Assessing the factors that influence the adoption of healthcare wearables by the older population using an extended PMT model. Technol. Soc..

[96] Nishant R., Srivastava S.C., Teo T.S.H. (2019). Using polynomial modeling to understand service quality in E-government websites. MIS Q..

[97] Sandberg J., Alvesson M. (2021). Meanings of theory: clarifying theory through typification. J. Manag. Stud..

