# Supplementary material for: Factors influencing technology use among low-income older adults: A systematic review
Source: Heliyon. 2023 Sep 13;9(9):e20111. doi: 10.1016/j.heliyon.2023.e20111 (PMC10559849; doi:10.1016/j.heliyon.2023.e20111)
Supplement: Multimedia component 1 [file mmc1.docx]

**Title: Factors influencing technology use among low-income older adults: a systematic review**

| **Section and Topic** | **Item #** | **Checklist item** | **Location where item is reported** |
| --- | --- | --- | --- |
| **TITLE** | | |  |
| Title | 1 | Identify the report as a systematic review. | Specified in Page 1, title “Factors influencing technology use among low-income older adults: a systematic review” |
| **ABSTRACT** | | |  |
| Abstract | 2 | See the PRISMA 2020 for Abstracts checklist. | The PRISMA 2020 Abstract checklist included 12 items to be reported in the Abstract. However, due to word count, only Item 1,2,8, 9 and 10 were stated in the abstract (Page 1) and the remaining items were reported in the other sections of the report. |
| **INTRODUCTION** | | |  |
| Rationale | 3 | Describe the rationale for the review in the context of existing knowledge. | Reported in Page 1-2, Section 1, Introduction (paragraph 2 and 3) |
| Objectives | 4 | Provide an explicit statement of the objective(s) or question(s) the review addresses. | Reported in Page 2, Introduction (last paragraph) |
| **METHODS** | | |  |
| Eligibility criteria | 5 | Specify the inclusion and exclusion criteria for the review and how studies were grouped for the syntheses. | Reported in Page 3, Section 3.1.2 (paragraph 1) |
| Information sources | 6 | Specify all databases, registers, websites, organisations, reference lists and other sources searched or consulted to identify studies. Specify the date when each source was last searched or consulted. | Reported in Page 3, Section 3.1.1 (paragraph 1) |
| Search strategy | 7 | Present the full search strategies for all databases, registers and websites, including any filters and limits used. | Reported search strategy in Page 3, Section 3.1.1, and Table 1. Filters and limits were not applied. |
| Selection process | 8 | Specify the methods used to decide whether a study met the inclusion criteria of the review, including how many reviewers screened each record and each report retrieved, whether they worked independently, and if applicable, details of automation tools used in the process. | Reported in Page 3, Section 3.1.2 (paragraph 1). Methods and number of reviewers were indicated. |
| Data collection process | 9 | Specify the methods used to collect data from reports, including how many reviewers collected data from each report, whether they worked independently, any processes for obtaining or confirming data from study investigators, and if applicable, details of automation tools used in the process. | Reported in Page 5, Section 3.2 (paragraph 1). No automation tools were used in the process. |
| Data items | 10a | List and define all outcomes for which data were sought. Specify whether all results that were compatible with each outcome domain in each study were sought (e.g. for all measures, time points, analyses), and if not, the methods used to decide which results to collect. | Reported definition of use of technology (outcome) in Page 2, Section 1.1.2, and methods in Section 3.2. |
|  | 10b | List and define all other variables for which data were sought (e.g. participant and intervention characteristics, funding sources). Describe any assumptions made about any missing or unclear information. | Reported definition of other key terminologies in Page 2, Section 1.1, and 1.1.1. |
| Study risk of bias assessment | 11 | Specify the methods used to assess risk of bias in the included studies, including details of the tool(s) used, how many reviewers assessed each study and whether they worked independently, and if applicable, details of automation tools used in the process. | Reported methods for quality assessment in Page 3, Section 3.1.2. |
| Effect measures | 12 | Specify for each outcome the effect measure(s) (e.g. risk ratio, mean difference) used in the synthesis or presentation of results. | Reported in Page 5, Section 3.2, “the odds ratios or percentages; p-value of regression analysis; positive or negative impact” |
| Synthesis methods | 13a | Describe the processes used to decide which studies were eligible for each synthesis (e.g. tabulating the study intervention characteristics and comparing against the planned groups for each synthesis (item #5)). | Reported in Page 3, Section 3.1.2 |
|  | 13b | Describe any methods required to prepare the data for presentation or synthesis, such as handling of missing summary statistics, or data conversions. | Not applicable−no statistical synthesis involved |
|  | 13c | Describe any methods used to tabulate or visually display results of individual studies and syntheses. | Reported in Page 5, Section 3.2 (paragraph 2) |
|  | 13d | Describe any methods used to synthesize results and provide a rationale for the choice(s). If meta-analysis was performed, describe the model(s), method(s) to identify the presence and extent of statistical heterogeneity, and software package(s) used. | Reported in Page 5, Section 3.2 (paragraph 2) |
|  | 13e | Describe any methods used to explore possible causes of heterogeneity among study results (e.g. subgroup analysis, meta-regression). | Not applicable to the objective of this study−no statistical synthesis involved |
|  | 13f | Describe any sensitivity analyses conducted to assess robustness of the synthesized results. | Not applicable to the objective of this study−not a meta-analysis, no statistical synthesis involved |
| Reporting bias assessment | 14 | Describe any methods used to assess risk of bias due to missing results in a synthesis (arising from reporting biases). | Not applicable to the objective of this study− no statistical synthesis involved |
| Certainty assessment | 15 | Describe any methods used to assess certainty (or confidence) in the body of evidence for an outcome. | Reported methods for quality assessment in Page 3, Section 3.1.2. |
| **RESULTS** | | |  |
| Study selection | 16a | Describe the results of the search and selection process, from the number of records identified in the search to the number of studies included in the review, ideally using a flow diagram. | Reported in Page 4, Fig. 1. and Page 5, Section 4.1. |
|  | 16b | Cite studies that might appear to meet the inclusion criteria, but which were excluded, and explain why they were excluded. | Reasons for exclusions were reported in Page 4 (flow diagram “Reports excluded”). The numbers of study excluded for each reason was indicated but studies were not cited. |
| Study characteristics | 17 | Cite each included study and present its characteristics. | Reported in Page 5, Section 4.1 and Page 20-21, Appendix A (Table A.1). |
| Risk of bias in studies | 18 | Present assessments of risk of bias for each included study. | Reported in Page 5, Section 4.2 (last sentence). |
| Results of individual studies | 19 | For all outcomes, present, for each study: (a) summary statistics for each group (where appropriate) and (b) an effect estimate and its precision (e.g. confidence/credible interval), ideally using structured tables or plots. | Reported in Page 22-26, Appendix B, Table B.1, Table B.2, Page 27, Appendix C, Table C.1. |
| Results of syntheses | 20a | For each synthesis, briefly summarise the characteristics and risk of bias among contributing studies. | Reported characteristics of contributing studies in Page 5, Section 4.2. |
|  | 20b | Present results of all statistical syntheses conducted. If meta-analysis was done, present for each the summary estimate and its precision (e.g. confidence/credible interval) and measures of statistical heterogeneity. If comparing groups, describe the direction of the effect. | Reported results in Page 6, Section 4.4, Page 7, Table 3, Page 7-15, Section 5-5.3. |
|  | 20c | Present results of all investigations of possible causes of heterogeneity among study results. | Not applicable to the objective of this study− no statistical synthesis involved |
|  | 20d | Present results of all sensitivity analyses conducted to assess the robustness of the synthesized results. | Not applicable to the objective of this study− no statistical synthesis involved |
| Reporting biases | 21 | Present assessments of risk of bias due to missing results (arising from reporting biases) for each synthesis assessed. | Not applicable to the objective of this study− no statistical synthesis involved |
| Certainty of evidence | 22 | Present assessments of certainty (or confidence) in the body of evidence for each outcome assessed. | Not applicable to the objective of this study− no statistical synthesis involved |
| **DISCUSSION** | | |  |
| Discussion | 23a | Provide a general interpretation of the results in the context of other evidence. | Reported in Page 15-18, Section 6-6.6. |
|  | 23b | Discuss any limitations of the evidence included in the review. | Reported in Page 19, Section 8. |
|  | 23c | Discuss any limitations of the review processes used. | Reported in Page 19, Section 8. |
|  | 23d | Discuss implications of the results for practice, policy, and future research. | Reported in Page 15-18, Section 6-6.6. |
| **OTHER INFORMATION** | | |  |
| Registration and protocol | 24a | Provide registration information for the review, including register name and registration number, or state that the review was not registered. | Reported in Page 3, Section 3.1. |
|  | 24b | Indicate where the review protocol can be accessed, or state that a protocol was not prepared. | Reported in Page 3, Section 3.1. |
|  | 24c | Describe and explain any amendments to information provided at registration or in the protocol. | Not applicable−no amendment of protocol involved |
| Support | 25 | Describe sources of financial or non-financial support for the review, and the role of the funders or sponsors in the review. | Reported in Page 20 (Acknowledgement) |
| Competing interests | 26 | Declare any competing interests of review authors. | Reported in Page 20 (Declaration of competing interest) |
| Availability of data, code and other materials | 27 | Report which of the following are publicly available and where they can be found: template data collection forms; data extracted from included studies; data used for all analyses; analytic code; any other materials used in the review. | Reported in Page 20 (Data availability) |

*From:*  Page MJ, McKenzie JE, Bossuyt PM, Boutron I, Hoffmann TC, Mulrow CD, et al. The PRISMA 2020 statement: an updated guideline for reporting systematic reviews. BMJ 2021;372:n71. doi: 10.1136/bmj.n71

For more information, visit: <http://www.prisma-statement.org/>
